# Multi-timescale analysis of a metabolic network in synthetic biology: a kinetic model for 3-hydroxypropionic acid production via beta-alanine

**DOI:** 10.1007/s00285-017-1189-3

**Published:** 2017-11-20

**Authors:** Mohit P. Dalwadi, John R. King, Nigel P. Minton

**Affiliations:** 1 Synthetic Biology Research Centre, University of Nottingham, University Park, Nottingham, NG7 2RD UK; 2 School of Mathematical Sciences, University of Nottingham, University Park, Nottingham, NG7 2RD UK

**Keywords:** Asymptotic analysis, Reaction kinetics, Synthetic biology, Metabolic pathways, 34E10, 92C45

## Abstract

A biosustainable production route for 3-hydroxypropionic acid (3HP), an important platform chemical, would allow 3HP to be produced without using fossil fuels. We are interested in investigating a potential biochemical route to 3HP from pyruvate through $\beta$-alanine and, in this paper, we develop and solve a mathematical model for the reaction kinetics of the metabolites involved in this pathway. We consider two limiting cases, one where the levels of pyruvate are never replenished, the other where the levels of pyruvate are continuously replenished and thus kept constant. We exploit the natural separation of both the time scales and the metabolite concentrations to make significant asymptotic progress in understanding the system without resorting to computationally expensive parameter sweeps. Using our asymptotic results, we are able to predict the most important reactions to maximize the production of 3HP in this system while reducing the maximum amount of the toxic intermediate compound malonic semi-aldehyde present at any one time, and thus we are able to recommend which enzymes experimentalists should focus on manipulating.

## Introduction

3-hydroxypropionic acid (3HP) can be used to produce many other valuable chemicals, such as acrylic acid, 1,3-propanediol, and biodegradable polyesters (Werpy et al. 2004; Jiang et al. 2009). 3HP can be derived from biological sources and, as current industrial methods to produce acrylic acid involve fossil fuels, a production route through 3HP provides a biosustainable alternative. Although an *in vivo* production line in a bacterial or fungal host is the eventual industrial target for 3HP, performing *in vitro* experiments is an important prior step. The controlled nature of *in vitro* experiments allows for more systematic deductions to be made, and hence *in vitro* experiments can highlight potential roadblocks that may be more difficult to analyse in a complex *in vivo* system. Mathematical modelling allows for further systematic progress to be made, and can reduce the experimental parameter space that needs to be searched.

**Fig. 1:**
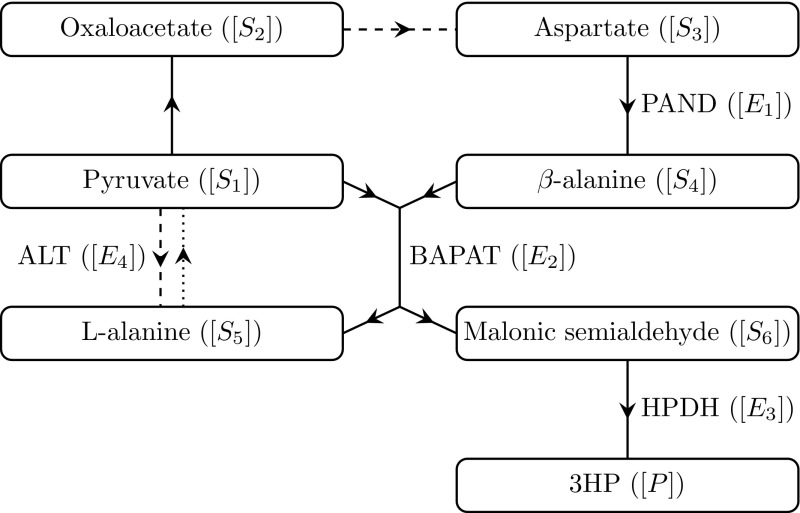
A schematic network diagram for the pathway we consider in this paper, where arrows denote the direction of the reactions. The dashed lines denote reactions which additionally require GLU ($[R_1]$) and produce AKG ($[R_2]$), and the dotted lines denote reactions which additionally require AKG and produce GLU. If we consider the enzyme mechanics for a reaction, we include the name of that enzyme next to the reaction arrow

In Kumar et al. (2013), three thermodynamically feasible pathways from pyruvate to 3HP are suggested. We are interested here in mathematically modelling the route through aspartate and $\beta$-alanine; this is the synthetic pathway successfully introduced to *Saccharomyces cerevisiae* in Borodina et al. (2015). Thus, we consider the reactions 1a
1b
1c
1d
1e
1f and we show a schematic representation of this pathway in Fig. 1.

Here, we do not consider enzyme mechanics for the first two reactions in (1). This is because pyruvate and oxaloacetate are required for the citric acid cycle, and we are not interested in altering any enzymatic processes that may significantly change this important metabolic cycle. Thus, the enzyme kinetics for the latter four reactions are the most appropriate targets for enhancement, and we therefore include these.

We note that (1e), the reaction between l-alanine and pyruvate, is not necessary for a connected pathway between pyruvate and 3HP. However, as (1e) recycles l-alanine and $\alpha$-ketoglutarate (AKG) back into pyruvate and glutamic acid (GLU), used in (1a) and (1b), respectively, the inclusion of (1e) may enhance the efficiency of the pathway. In Borodina et al. (2015), it was hypothesized that (1e) may have an effect on the overall 3HP production, and we investigate this hypothesis here. More generally, we are interested in determining which of the reactions are the most important for 3HP production. Additionally, malonic semialdehyde is a toxic compound, and so we will also look for ways to reduce the amount of malonic semialdehyde present in the system. To summarize, the general goal of this system, so far as possible, is to maximize the 3HP produced whilst minimizing the maximum level of malonic semialdehyde present in the system.

We will assume that there are enough molecules of each metabolite to use the law of mass action and that the entire system is well-mixed and thus spatially independent. Moreover, in any given reaction, we neglect any inhibitory effects from metabolites not involved in the reaction and from any products of the reaction. We do so to limit the number of parameters in the model, and to enable us to make analytic progress, thus allowing us to develop physical insight into the system. Additionally, the production levels of pyruvate within the system will vary in time. We consider two limiting cases, pyruvate being a finite resource which is never replenished in the first, but an infinite resource held at a constant concentration in the second. Whilst the actual levels of pyruvate will, in practice, fall somewhere between these two cases, it is useful to analyse both in detail: exploring and comparing these two extreme cases will provide insight into the general pathway kinetics in intermediate cases.

As there are many parameters involved in this problem, a fully experimental approach would be very time consuming. For the same reason, investigating its mathematical model using a purely numerical approach would also be a protracted process, albeit shorter than the fully experimental one. We perform an asymptotic analysis (see, for example, Kevorkian and Cole 2013; Hinch 1991; O’Malley Jr (2012)) to investigate our model, thus enhancing our physical insight into the underlying system and enabling us to determine how the concentrations vary as functions of the experimental parameters. Due to this approach, exact knowledge of each kinetic parameter is not required, just an estimate of the order of magnitude of each parameter.

We introduce a mathematical model to describe the nonlinear reaction kinetics in §2. We solve this for non-replenished and continuously replenished pyruvate in §3 and §4, respectively, where we give both numerical and asymptotic solutions to describe the system behaviour. We also consider the problem of general pyruvate production in “Appendix C”, where we derive asymptotic solutions in one time regime for the metabolite concentrations at leading order in terms of the pyruvate in the system. We finish by discussing our results and comparing the two regimes in §5, where we also suggest future avenues of inquiry that follow from this work.

## Model description

We assume that the reaction kinetics are governed by the law of mass action, yielding the system

2a $$\begin{aligned} \frac{\mathrm {d} [S_1]}{\mathrm {d} \tau }&= -k_1[S_1]- k_5[S_1][S_4][E_2]+ k_{-5}[C_2]+ k_8[C_4]- k_{-8}[S_1][R_1][E_4], \end{aligned}$$

2b $$\begin{aligned} \frac{\mathrm {d} [S_2]}{\mathrm {d} \tau }&= k_1[S_1]- k_2[S_2][R_1], \end{aligned}$$

2c $$\begin{aligned} \frac{\mathrm {d} [S_3]}{\mathrm {d} \tau }&= k_2[S_2][R_1]- k_{3}[S_3][E_1]+ k_{-3}[C_1], \end{aligned}$$

2d $$\begin{aligned} \frac{\mathrm {d} [S_4]}{\mathrm {d} \tau }&= k_4[C_1]- k_5[S_4][S_1][E_2]+ k_{-5}[C_2], \end{aligned}$$

2e $$\begin{aligned} \frac{\mathrm {d} [S_5]}{\mathrm {d} \tau }&= k_6[C_2]- k_{7}[S_5][R_2][E_4]+ k_{-7}[C_4], \end{aligned}$$

2f $$\begin{aligned} \frac{\mathrm {d} [S_6]}{\mathrm {d} \tau }&= k_6[C_2]- k_9[S_6][E_3]+ k_{-9}[C_3], \end{aligned}$$

2g $$\begin{aligned} \frac{\mathrm {d} [R_1]}{\mathrm {d} \tau }&= - k_2[S_2][R_1]+ k_8[C_4]- k_{-8}[S_1][R_1][E_4], \end{aligned}$$

2h $$\begin{aligned} \frac{\mathrm {d} [R_2]}{\mathrm {d} \tau }&= k_2[S_2][R_1]- k_{7}[S_5][R_2][E_4]+ k_{-7}[C_4], \end{aligned}$$

2i $$\begin{aligned} \frac{\mathrm {d} [E_1]}{\mathrm {d} \tau }&= -k_{3}[S_3][E_1]+ k_{-3}[C_1]+ k_4[C_1], \end{aligned}$$

2j $$\begin{aligned} \frac{\mathrm {d} [E_2]}{\mathrm {d} \tau }&= -k_5[S_4][S_1][E_2]+ k_{-5}[C_2]+ k_6[C_2], \end{aligned}$$

2k $$\begin{aligned} \frac{\mathrm {d} [E_3]}{\mathrm {d} \tau }&= -k_9[S_6][E_3]+ k_{-9}[C_3]+ k_{10}[C_3], \end{aligned}$$

2l $$\begin{aligned} \frac{\mathrm {d} [E_4]}{\mathrm {d} \tau }&= -k_{7}[S_5][R_2][E_4]+ k_{-7}[C_4]+ k_8[C_4]- k_{-8}[S_1][R_1][E_4], \end{aligned}$$

2m $$\begin{aligned} \frac{\mathrm {d} [C_1]}{\mathrm {d} \tau }&= k_{3}[S_3][E_1]- k_{-3}[C_1]- k_4[C_1], \end{aligned}$$

2n $$\begin{aligned} \frac{\mathrm {d} [C_2]}{\mathrm {d} \tau }&= k_5[S_4][S_1][E_2]- k_{-5}[C_2]- k_6[C_2], \end{aligned}$$

2o $$\begin{aligned} \frac{\mathrm {d} [C_3]}{\mathrm {d} \tau }&= k_9[S_6][E_3]- k_{-9}[C_3]- k_{10}[C_3], \end{aligned}$$

2p $$\begin{aligned} \frac{\mathrm {d} [C_4]}{\mathrm {d} \tau }&= k_{7}[S_5][R_2][E_4]- k_{-7}[C_4]- k_8[C_4]+ k_{-8}[S_1][R_1][E_4], \end{aligned}$$

2q $$\begin{aligned} \frac{\mathrm {d} [P]}{\mathrm {d} \tau }&= k_{10}[C_3]. \end{aligned}$$

All the variables introduced here are defined in Table 1, and the units and typical values for each kinetic parameter are given in Table 2. We model a scenario where each of the enzymes are introduced to a solution containing only pyruvate and glutamic acid, and this resulting mixture is instantaneously well-mixed. We assume that the initial concentration of pyruvate is $[S_1](0) = S_0$, where $S_0$ is around $1 \, \mathrm {mM}$. We will state the remaining initial conditions after discussing how we deal with uncertainty in the kinetic parameters.

**Table 1:** Dimensional and dimensionless variable definitions

Original variable	Description	Nondimensionalisation
$[S_1]$	Pyruvate	$[S_1]= S_0S_1$
$[S_2]$	Oxaloacetate	$[S_2]= S_0S_2$
$[S_3]$	Aspartate	$[S_3]= \varepsilon S_0S_3$
$[S_4]$	$\beta$-Alanine	$[S_4]= \varepsilon S_0S_4$
$[S_5]$	*L*-Alanine	$[S_5]= \varepsilon S_0S_5$
$[S_6]$	Malonic semialdehyde	$[S_6]= \varepsilon ^4 S_0S_6$
$[R_1]$	Glutamic acid (GLU)	$[R_1]= S_0R_1$
$[R_2]$	$\alpha$-Ketoglutarate (AKG)	$[R_2]= \varepsilon S_0R_2$
$[E_1]$	Aspartate decarboxylase (PAND)	$[E_1]= \varepsilon a_1S_0E_1$
$[E_2]$	$\beta$-Alanine-pyruvate aminotransferase (BAPAT)	$[E_2]= \varepsilon a_2S_0E_2$
$[E_3]$	3-Hydroxypropionate dehydrogenase (HPDH)	$[E_3]= \varepsilon a_3S_0E_3$
$[E_4]$	Alanine aminotransferase (ALT)	$[E_4]= \varepsilon a_4S_0E_4$
$[C_1]$	Aspartate decarboxylase complex (PAND$_c$)	$[C_1]= \varepsilon ^2 a_1S_0C_1$
$[C_2]$	$\beta$-Alanine-pyruvate aminotransferase complex (BAPAT$_c$)	$[C_2]= \varepsilon ^4 a_2S_0C_2$
$[C_3]$	3-Hydroxypropionate dehydrogenase complex (HPDH$_c$)	$[C_3]= \varepsilon ^5 a_3S_0C_3$
$[C_4]$	Alanine aminotransferase complex (ALT$_c$)	$[C_4]= \varepsilon ^3 a_4S_0C_4$
$[P]$	3-Hydroxypropionic acid (3HP)	$[P]= \varepsilon ^3 S_0P$
$\tau$	Time	$\tau = t/ k_1$

The dimensional metabolite concentrations are denoted with square brackets and have units of moles per volume, as does $S_0$

**Table 2:** Kinetic reaction rate parameters

Dimensional parameter	Organism	Parameter range
$k_1= 10^{-2} \, \mathrm {s}^{-1}$	*Saccharomyces cerevisiae* $^\mathrm{a}$	$8 \times 10^{-3}$ – $2 \times 10^{-1} \, \mathrm {s}^{-1} \, ^\mathrm{a,g}$
$k_2= 7 \times 10^{-5} \, \mathrm {mM}^{-1} \mathrm {s}^{-1}$	*Thermus thermophilus* $^\mathrm{b}$	$7 \times 10^{-5}$ – $1 \times 10^{-3} \, \mathrm {mM}^{-1} \mathrm {s}^{-1} \, ^\mathrm{b,h}$
$k_{3}= 10^{2} \, \mathrm {mM}^{-1} \mathrm {s}^{-1}$	*Escherichia coli* $^\mathrm{c}$	$1 \times 10^{1}$ – $1.6 \times 10^{3} \, \mathrm {mM}^{-1} \mathrm {s}^{-1} \, ^\mathrm{c,i,j}$
$k_{-3}= 2 \times 10^{1} \, \mathrm {s}^{-1}$	*Escherichia coli* $^\mathrm{c}$	$1 \times 10^{0}$ – $2 \times 10^{2} \, \mathrm {s}^{-1} \, ^\mathrm{c,i,j}$
$k_4= 1 \, \mathrm {s}^{-1}$	*Escherichia coli* $^\mathrm{c}$	$2 \times 10^{-1}$ – $2 \times 10^{1} \, \mathrm {s}^{-1} \, ^\mathrm{c,i,j}$
$k_5= 4 \times 10^{-2} \, \mathrm {mM}^{-2} \mathrm {s}^{-1}$	*Bacillus cereus* $^\mathrm{d}$	$7 \times 10^{-3}$ – $2 \times 10^{-1} \, \mathrm {mM}^{-2} \mathrm {s}^{-1} \, ^\mathrm{d,k}$
$k_{-5}= 80 \, \mathrm {s}^{-1}$	*Bacillus cereus* $^\mathrm{d}$	$1 \times 10^{1}$ – $5 \times 10^{2} \, \mathrm {s}^{-1} \, ^\mathrm{d,k}$
$k_6= 1.5 \, \mathrm {s}^{-1}$	*Bacillus cereus* $^\mathrm{d}$	$1 \times 10^{0}$ – $4 \times 10^{0} \, \mathrm {s}^{-1} \, ^\mathrm{d,k}$
$k_{7}= 3 \times 10^{-2} \, \mathrm {mM}^{-2} \mathrm {s}^{-1}$	*Pyrococcus furiosus* $^\mathrm{e}$	$1 \times 10^{-2}$ – $2 \times 10^{0} \, \mathrm {mM}^{-2} \mathrm {s}^{-1} \, ^\mathrm{e,l}$
$k_{-7}= 10^{2} \, \mathrm {s}^{-1}$	*Pyrococcus furiosus* $^\mathrm{e}$	$5 \times 10^{0}$ – $6 \times 10^{2} \, \mathrm {s}^{-1} \, ^\mathrm{e,l}$
$k_8= 10^{2} \, \mathrm {s}^{-1}$	*Pyrococcus furiosus* $^\mathrm{e}$	$5 \times 10^{0}$ – $6 \times 10^{2} \, \mathrm {s}^{-1} \, ^\mathrm{e,l}$
$k_{-8}= 4 \times 10^{-2} \, \mathrm {mM}^{-2} \mathrm {s}^{-1}$	*Pyrococcus furiosus* $^\mathrm{e}$	$1 \times 10^{-2}$ – $1 \times 10^{0} \, \mathrm {mM}^{-2} \mathrm {s}^{-1} \, ^\mathrm{e,l}$
$k_9= 10^{2} \, \mathrm {mM}^{-1} \mathrm {s}^{-1}$	*Metallosphaera sedula* $^\mathrm{f}$	$5 \times 10^{1}$ – $2\times 10^{2} \, \mathrm {mM}^{-1} \mathrm {s}^{-1} \, ^\mathrm{f}$
$k_{-9}= 5 \, \mathrm {s}^{-1}$	*Metallosphaera sedula* $^\mathrm{f}$	$1 \times 10^{0}$ – $1 \times 10^{1} \, \mathrm {s}^{-1} \, ^\mathrm{f}$
$k_{10}= 30 \, \mathrm {s}^{-1}$	*Metallosphaera sedula* $^\mathrm{f}$	$1 \times 10^{0}$ – $1 \times 10^{2} \, \mathrm {s}^{-1} \, ^\mathrm{f,m}$

Dimensionless parameter		
$$\bar{k}_2= k_2S_0/ (\varepsilon k_1) = 0.7$$		
$$\bar{k}_{3}= k_{3}a_1S_0\varepsilon ^2 / k_1= a_1$$		
$$\bar{k}_{-3}= k_{-3}a_1\varepsilon ^2 / k_1= 0.2 a_1$$		
$$\bar{k}_4= k_4a_1\varepsilon / k_1= a_1$$		
$$\bar{k}_{5}= k_5a_2S_0^2 / k_1= 4 a_2$$		
$$\bar{k}_{-5}= k_{-5}a_2\varepsilon ^2 / k_1= 0.8 a_2$$		
$$\bar{k}_{6}= k_6a_2\varepsilon / k_1= 1.5 a_2$$		
$$\bar{k}_{7}= k_{7}a_4S_0^2 / k_1= 3 a_4$$		
$$\bar{k}_{-7}= k_{-7}a_4\varepsilon ^2 / k_1= a_4$$		
$$\bar{k}_8= k_8a_4\varepsilon ^2/ k_1= a_4$$		
$$\bar{k}_{-8}= k_{-8}a_4S_0^2/ k_1= 4 a_4$$		
$$\bar{k}_{9}= k_9a_3S_0\varepsilon ^2 / k_1= a_3$$		
$$\bar{k}_{-9}= k_{-9}a_3\varepsilon / k_1= 5 a_3$$		
$$\bar{k}_{10}= k_{10}a_3\varepsilon ^2/ k_1= 0.3 a_3$$		

In general, it is only ratios of kinetic parameters that are known for a given reaction, as substrate binding to an enzyme is too quick to accurately measure the reaction rate. We therefore choose parameter values (using the ratios given in the references) that lead to the enzyme complexes being formed over a time period that is much shorter than the time period over which the metabolite concentrations change. This, experimental uncertainty, and differences in enzymes in different organisms lead to a large possible range for the parameter values. In scaling the kinetic parameters with $S_0$ and powers of $\varepsilon$, we have used the values $S_0= 1 \mathrm {mM}$, $\varepsilon = 10^{-2}$. The values of $k_1$ and $k_2$ are chosen to be effective reaction rates as we are not interested in modifying native enzymes. Thus, $k_1$ is the ratio of the PYC turnover number to the Michaelis constant for pyruvate multiplied by an estimated concentration of PYC, chosen to be around $1\,\upmu \mathrm {M}$. $k_2$ is the ratio of the AAT turnover number to the product of the Michaelis constants for oxaloacetate and GLU, multiplied by an estimated concentration of AAT, chosen to be around $0.1\,\upmu \mathrm {M}$. The references are: $^\mathrm{a}$ Branson et al. (2002), $^\mathrm{b}$ Nobe et al. (1998), $^\mathrm{c}$ Ramjee et al. (1997), $^\mathrm{d}$ Nakano et al. (1977), $^\mathrm{e}$ Ward et al. (2000), $^\mathrm{f}$ Kockelkorn and Fuchs (2009), $^\mathrm{g}$ Jitrapakdee et al. (2007), $^\mathrm{h}$ Yagi et al. (1982), $^\mathrm{i}$ Chopra et al. (2002), $^\mathrm{j}$ Williamson and Brown (1979), $^\mathrm{k}$ Hayaishi et al. (1961), $^\mathrm{l}$ Umemura et al. (1994), $^\mathrm{m}$ Berg et al. (2007)

We see in Table 2 that estimates for the kinetic parameters can vary over three orders of magnitude between different organisms. To investigate how the system behaves as these parameters vary we first form dimensionless variables, scaling each dimensional metabolite concentration with $$S_0$$ and time with $$1/k_1$$ (the characteristic time of the first reaction between pyruvate and oxaloacetate). Moreover, we form dimensionless parameters by scaling the kinetic parameters with $$k_1$$ and the appropriate power of $$S_0$$. We then introduce the artificial small dimensionless parameter $$\varepsilon = 10^{-2}$$, and allow each dimensionless system parameter to be written as $$c \varepsilon ^{j}$$ where *c* is an $$ O (1)$$ parameter (between 0.1 and 10), and *j* is an integer. The dimensionless parameters in our system are given in the lower half of Table 2. This approach allows us to interrogate the system using an asymptotic analysis. Although (as always) there may theoretically be an issue with this method in equating terms with the same powers of $$\varepsilon $$ when large (or small) $$ O (1)$$ parameters are multiplied together, we will show that our asymptotic and numerical results show excellent agreement, and thus the approach is reliable for this system.

For the initial conditions, we assume that the initial levels of pyruvate and of glutamic acid are of comparable size and, to mimic the environment within a cell, that these concentrations are much larger than the initial concentrations of each enzyme. Additionally, we assume that the initial concentrations of each enzyme are of comparable size. Although enzyme-to-substrate levels will vary, a reasonable assumption is that this ratio is around 1:100 and thus of $$ O (\varepsilon )$$ for our problem (Albe et al. 1990). Thus, recalling that $$[S_1](0) = S_0$$, we also have $$[R_1](0) = \alpha S_0$$, $$[E_1](0) = \varepsilon a_1S_0$$, $$[E_2](0) = \varepsilon a_2S_0$$, $$[E_3](0) = \varepsilon a_3S_0$$, and $$[E_4](0) = \varepsilon a_4S_0$$, noting that the remaining metabolite concentrations start at zero. The dimensionless parameters $$\alpha $$, $$a_1$$, $$a_2$$, $$a_3$$, and $$a_4$$ are of $$ O (1)$$. These conditions will allow us to investigate how the system changes as the initial conditions of the system vary when we perform our asymptotic analysis. From the initial conditions and (2i–2p), we can immediately deduce that

3 $$\begin{aligned}{}[E_i] + [C_i] = [E_i](0), \end{aligned}$$

where $$i = 1, 2, 3, 4$$.

As we now have our dimensionless variables in terms of powers of $\varepsilon$, we can scale each concentration variable with the appropriate power of $\varepsilon$ to obtain the correct leading-order system for $t= O (1)$. This will allow us to immediately perform an asymptotic analysis for small $\varepsilon$. We give the correct scalings for these variables in Table 1, obtained by considering a dominant balance for each equation in the system and the non-zero initial conditions discussed above. We will perform a similar procedure to determine the correct asymptotic scalings over longer timescales, for which we will use effective ‘initial’ conditions obtained by asymptotic matching between timescales. The dimensionless form of (3) yields the relationships $E_1= 1 - \varepsilon C_1$, $E_2= 1 - \varepsilon ^3 C_2$, $E_3= 1 - \varepsilon ^4 C_3$, and $E_4= 1 - \varepsilon ^2 C_4$. Replacing each instance of $E_i$ (for $i \in \{1,2,3,4 \}$) with the appropriate function of $C_i$ allows us to reduce the dimension of the system of ODEs, accounting for (2i–2l). The remaining dimensionless system is

4a $$\begin{aligned} \dot{S_1}&= - S_1- \varepsilon ^2 \bar{k}_{5}S_1S_4\left( 1 - \varepsilon ^3C_2\right) + \varepsilon ^2 \bar{k}_{-5}C_2+ \varepsilon \bar{k}_8C_4\! - \varepsilon \bar{k}_{-8}S_1R_1\left( 1 - \varepsilon ^2 C_4\right) , \end{aligned}$$

4b $$\begin{aligned} \dot{S_2}&= S_1- \varepsilon \bar{k}_2S_2R_1, \end{aligned}$$

4c $$\begin{aligned} \varepsilon \dot{S_3}&= \varepsilon \bar{k}_2S_2R_1- \bar{k}_{3}S_3\left( 1 - \varepsilon C_1\right) + \bar{k}_{-3}C_1, \end{aligned}$$

4d $$\begin{aligned} \dot{S_4}&= \bar{k}_4C_1- \varepsilon \bar{k}_{5}S_1S_4\left( 1 - \varepsilon ^3C_2\right) + \varepsilon \bar{k}_{-5}C_2, \end{aligned}$$

4e $$\begin{aligned} \dot{S_5}&= \varepsilon ^2 \bar{k}_{6}C_2- \varepsilon ^2 \bar{k}_{7}S_5R_2\left( 1 - \varepsilon ^2 C_4\right) + \bar{k}_{-7}C_4, \end{aligned}$$

4f $$\begin{aligned} \varepsilon \dot{S_6}&= \bar{k}_{6}C_2- \bar{k}_{9}S_6\left( 1 - \varepsilon ^4 C_3\right) + \varepsilon \bar{k}_{-9}C_3, \end{aligned}$$

4g $$\begin{aligned} \dot{R_1}&= - \varepsilon \bar{k}_2S_2R_1+ \varepsilon \bar{k}_8C_4- \varepsilon \bar{k}_{-8}S_1R_1\left( 1 - \varepsilon ^2 C_4\right) , \end{aligned}$$

4h $$\begin{aligned} \dot{R_2}&= \bar{k}_2S_2R_1- \varepsilon ^2 \bar{k}_{7}S_5R_2\left( 1 - \varepsilon ^2 C_4\right) + \bar{k}_{-7}C_4, \end{aligned}$$

4i $$\begin{aligned} \varepsilon ^2 \dot{C_1}&=\bar{k}_{3}S_3\left( 1 - \varepsilon C_1\right) - \bar{k}_{-3}C_1- \varepsilon \bar{k}_4C_1, \end{aligned}$$

4j $$\begin{aligned} \varepsilon ^2 a_2\dot{C_2}&= \bar{k}_{5}S_1S_4\left( 1 - \varepsilon ^3C_2\right) - \bar{k}_{-5}C_2- \varepsilon \bar{k}_{6}C_2, \end{aligned}$$

4k $$\begin{aligned} \varepsilon ^2 a_3\dot{C_3}&= \bar{k}_{9}S_6\left( 1 - \varepsilon ^4 C_3\right) - \varepsilon \bar{k}_{-9}C_3- \bar{k}_{10}C_3, \end{aligned}$$

4l $$\begin{aligned} \varepsilon ^2 a_4\dot{C_4}&= \varepsilon ^2 \bar{k}_{7}S_5R_2\left( 1 - \varepsilon ^2 C_4\right) - \bar{k}_{-7}C_4- \bar{k}_8C_4+ \bar{k}_{-8}S_1R_1\left( 1 - \varepsilon ^2 C_4\right) , \end{aligned}$$

4m $$\begin{aligned} \dot{P}&= \bar{k}_{10}C_3, \end{aligned}$$

where the dimensionless parameters are defined in Table 2 and an overdot denotes $\mathrm {d}/\mathrm {d}t$. In the following two sections, we solve the system presented in (4) for non-replenished and constantly replenished pyruvate, respectively. The non-replenished case is governed by (4), and the replenished case by (4b–m), with (4a) replaced by $S_1\equiv 1$. For future reference, we note the immediate consequence of (4c) and (4i), that

5 $$\begin{aligned} \dot{S_3} + \varepsilon \dot{C_1}&= \bar{k}_2S_2R_1- \bar{k}_4C_1, \end{aligned}$$

which is a statement about the evolution of aspartate from oxaloacetate and towards PAND$_c$—(5) follows on dividing the sum of (4c) and (4i) by $\varepsilon$, and will furnish an additional leading-order equation in the limit $\varepsilon \rightarrow 0$, in which (4c) and (4i) as they stand lead to a duplication of information.

## Non-replenished pyruvate

### Numerical results

**Fig. 2:**
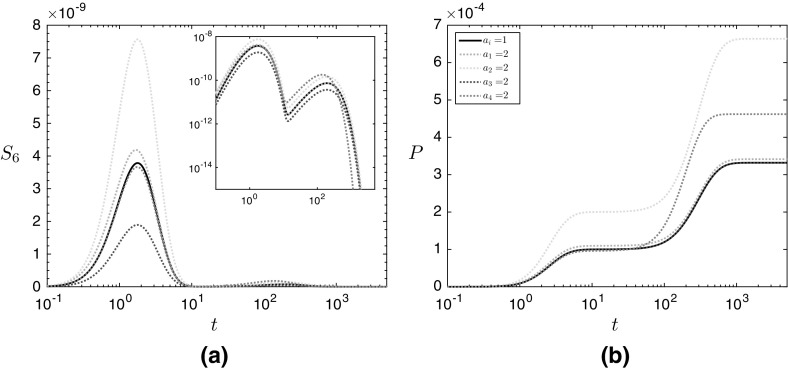
Numerical solutions in the non-replenished pyruvate case for the dynamic concentrations of **a** malonic semialdehyde and **b** 3HP. We use the parameter values given in Table 2 for all simulations, and initial dimensionless enzyme concentrations of 1 unless specified in the legend. The solid black line corresponds to the reference simulation where $a_i = 1$ for $i = 1, 2, 3, 4$

We solve the system (4) numerically, using ode15s in MATLAB. Our asymptotic solutions will reveal that the system is stiff, and our choice of numerical approach reflects this. We use the parameter values given in Table 2, and see that there are two important timescales, where $t= O (1)$ and $t= O (1/\varepsilon )$ (recalling that $\varepsilon = 10^{-2}$), respectively (Fig. 2). Over each of these timescales, the levels of malonic semialdehyde rise then fall, and the levels of 3HP rise to a plateau. Additionally, the levels of malonic semialdehyde and 3HP can vary significantly when the initial concentration of different enzymes is increased (over-expression). We model the effect of over-expressing a particular enzyme by starting the simulation with twice the levels of that enzyme compared to the black reference line in Fig. 2. We emphasize that the parameter values we give are not likely to be exact but, as we will later derive solutions to our system as functions of these parameters, we will be able to determine which reactions are the most important to the system by analyzing the form of these solutions without knowledge of the exact values that these parameters take.

We now discuss how the over-expression of a given enzyme affects the system. A schematic for the metabolic network is given in Fig. 1. Increasing the levels of PAND has a positive but small effect on the levels of both malonic semialdehyde and 3HP. Over-expressing BAPAT has a much larger effect, significantly increasing the levels of both malonic semialdehyde and 3HP. In contrast, we see that over-expressing HPDH does not appear to have any effect on the levels of 3HP and, in fact, significantly decreases the maximum amount of malonic semialdehyde in the system. Finally, we see that over-expressing ALT has a very small negative effect on the levels of malonic semialdehyde, and a significant positive effect on the levels of 3HP.

In the next section, we explore the system (4) using an asymptotic analysis. This will allow us to determine how the system depends on the dimensionless parameters, and thus to explore the experimental parameter space without resorting to computationally expensive parameter sweeps.

### Asymptotic structure

We now investigate the system (4) by exploiting the small parameter, $\varepsilon$, using an asymptotic analysis. There are two main asymptotic regions of interest in time, where $t= O (1)$ and $t= O (1/\varepsilon )$, which we refer to as medium and long time, respectively (Fig. 2).

When $t= O (1)$, the system is governed by the release of pyruvate and GLU through the main pathway to 3HP and towards the production of l-alanine. When $t= O (1/\varepsilon )$, the levels of pyruvate have diminished, and the system is governed by the degradation of oxaloacetate with GLU into aspartate and AKG. Additionally, the l-alanine produced when $t= O (1)$ is now converted back into pyruvate which, although only a small amount, is enough to have a leading-order effect on the reaction with $\beta$-alanine, and thus a leading-order effect on the amount of 3HP produced. Thus, the l-alanine acts as a store of pyruvate over $t= O (1)$, and this is released over $t= O (1/\varepsilon )$. In the next section, we solve the leading-order versions of the system (4) in each of these asymptotic regions.

### Asymptotic solutions

The early-time behaviour of the system is given in “Appendix A”, obtained by Taylor expanding the initial conditions as $t\rightarrow 0^{+}$. The more interesting behaviour for malonic semialdehyde and 3HP production occurs for medium and long time, and we now consider the system dynamics over these timescales in turn.

#### Medium time: $t= O (1)$

As $\varepsilon \rightarrow 0$, the full system (4) becomes the differential-algebraic system

6a $$\begin{aligned}&\dot{S_1} = - S_1, \quad \dot{S_2} = S_1, \quad \dot{S_3} = \bar{k}_2S_2R_1- \bar{k}_4C_1, \quad \dot{S_4} = \bar{k}_4C_1, \quad \dot{S_5} = \bar{k}_{-7}C_4, \nonumber \\&\dot{R_1} = 0, \quad \dot{R_2} = \bar{k}_2S_2R_1+ \bar{k}_{-7}C_4, \quad \dot{P} = \bar{k}_{10}C_3, \end{aligned}$$

6b $$\begin{aligned}&\bar{k}_{9}S_6= \bar{k}_{6}C_2, \quad \bar{k}_{-3}C_1=\bar{k}_{3}S_3, \quad \bar{k}_{-5}C_2= \bar{k}_{5}S_1S_4, \quad \bar{k}_{10}C_3= \bar{k}_{9}S_6, \nonumber \\&\left( \bar{k}_{-7}+ \bar{k}_8\right) C_4= \bar{k}_{-8}S_1R_1, \end{aligned}$$

on using (5).

We solve the leading-order system (6) explicitly as $\varepsilon \rightarrow 0$, and the solutions are given by

7a $$\begin{aligned} S_1&= e^{-t}, \end{aligned}$$

7b $$\begin{aligned} S_2&= 1 - e^{-t}, \end{aligned}$$

7c $$\begin{aligned} S_3&= \beta _3\left( 1 - \dfrac{\omega e^{-t} - e^{-\omega t}}{\omega - 1}\right) , \end{aligned}$$

7d $$\begin{aligned} S_4&= \beta _4\left( t+ \dfrac{\omega ^2 e^{-t} - e^{-\omega t} + 1 - \omega ^2}{\omega (\omega - 1)}\right) , \end{aligned}$$

7e $$\begin{aligned} S_5&= \beta _5\left( 1 - e^{-t} \right) , \end{aligned}$$

7f $$\begin{aligned} S_6&= \beta _6\left( t+ \dfrac{\omega ^2 e^{-t} - e^{-\omega t} + 1 - \omega ^2}{\omega (\omega - 1)}\right) e^{-t}, \end{aligned}$$

7g $$\begin{aligned} R_1&= \alpha , \end{aligned}$$

7h $$\begin{aligned} R_2&= \beta _4\left( t+ e^{-t} - 1 \right) + \beta _5\left( 1 - e^{-t} \right) , \end{aligned}$$

7i $$\begin{aligned} C_1&= \gamma _1\left( 1 - \dfrac{\omega e^{-t} - e^{-\omega t}}{\omega - 1}\right) , \end{aligned}$$

7j $$\begin{aligned} C_2&= \gamma _2\left( t+ \dfrac{\omega ^2 e^{-t} - e^{-\omega t} + 1 - \omega ^2}{\omega (\omega - 1)}\right) e^{-t}, \end{aligned}$$

7k $$\begin{aligned} C_3&= \gamma _3\left( t+ \dfrac{\omega ^2 e^{-t} - e^{-\omega t} + 1 - \omega ^2}{\omega (\omega - 1)}\right) e^{-t}, \end{aligned}$$

7l $$\begin{aligned} C_4&= \gamma _4e^{-t}, \end{aligned}$$

7m $$\begin{aligned} P&= \lambda \left( \left( \dfrac{e^{-\omega t}}{\omega \left( \omega ^2 - 1 \right) } - t+ \dfrac{1}{\omega } - \dfrac{\omega e^{-t}}{2\left( \omega - 1 \right) }\right) e^{-t} + \dfrac{\omega }{2 \left( \omega + 1\right) } \right) , \end{aligned}$$

where we define the parameters

8 $$\begin{aligned} \beta _3&= \alpha \bar{k}_2/\omega , \quad \beta _4= \alpha \bar{k}_2, \quad \beta _5= \alpha \bar{k}_{-7}\bar{k}_{-8}/(\bar{k}_{-7}+ \bar{k}_8), \quad \beta _6= \alpha \bar{k}_2\bar{k}_{5}\bar{k}_{6}/(\bar{k}_{-5}\bar{k}_{9}),\nonumber \\ \gamma _1&= \alpha \bar{k}_2/ \bar{k}_4, \quad \gamma _2= \alpha \bar{k}_2\bar{k}_{5}/\bar{k}_{-5}, \quad \gamma _3= \alpha \bar{k}_2\bar{k}_{5}\bar{k}_{6}/(\bar{k}_{-5}\bar{k}_{10}), \nonumber \\ \gamma _4&= \alpha \bar{k}_{-8}/(\bar{k}_{-7}+ \bar{k}_8), \lambda = \alpha \bar{k}_2\bar{k}_{5}\bar{k}_{6}/\bar{k}_{-5}, \quad \omega = \bar{k}_{3}\bar{k}_4/ \bar{k}_{-3}. \end{aligned}$$

From Table 2, we see that the parameter $\omega = \bar{k}_{3}\bar{k}_4/ \bar{k}_{-3}$ is associated with (1c), the reaction from aspartate to $\beta$-alanine. That is, $\omega$ is proportional to both the ratio of the forward to backward reaction rate constants in (1c) and to the initial concentration of PAND, the enzyme that controls (1c). Thus, from the prevalence of $\omega$ in the solution (7), we see that (1c) affects the timing of the system when $t= O (1)$, and the other reactions control the concentration levels of the metabolites. For later analysis, we additionally note that the parameter grouping $\bar{k}_{5}\bar{k}_{6}/\bar{k}_{-5}$ is associated with (1d), the reaction from $\beta$-alanine and pyruvate to l-alanine and malonic semialdehyde.

**Fig. 3:**
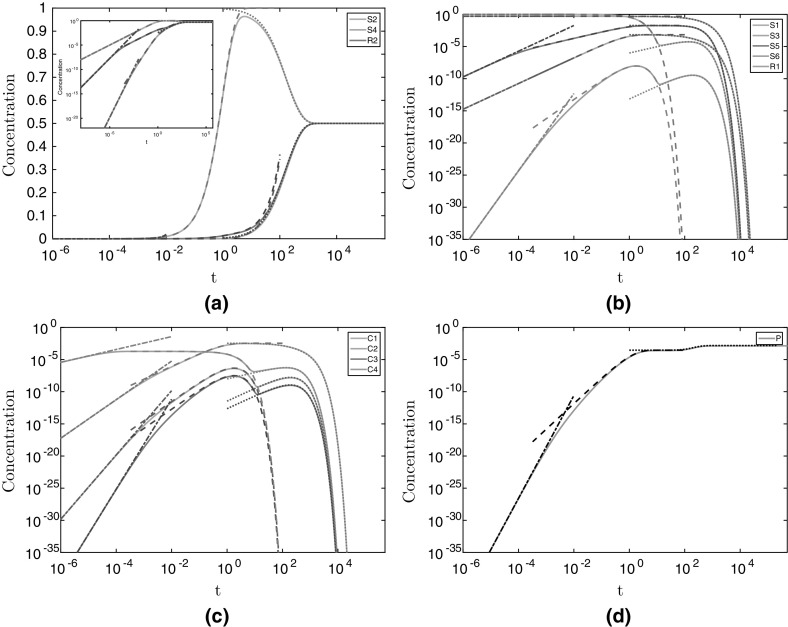
The numerical and asymptotic solutions for the metabolite concentrations in the non-replenished pyruvate case. The solid light lines denote the numerical solutions, and the broken darker lines denote the asymptotic solutions. Dashed lines represent the $t = O (1)$ solutions given in (7), dash-dotted lines represent the early-time solutions given in “Appendix A”, and dotted lines represent the late time solutions given in (11, B2), and (B4). We use parameter values $\alpha = 0.5$, $a_1= 1$, $a_2= 1$, $a_3= 1$, and $a_4= 1$. We have split the metabolite concentrations into **a** the substrates that tend to a non-zero constant value for large time, **b** the substrates which tend to zero for large time, **c** the enzyme complexes, and **d** the product. We plot $([S_i],[R_i],[C_i],[P])/S_0$

We note there is an implicit assumption that $\omega \ne 1$ in writing (7), but the apparent singularities are removable in this limit and, as such, they do not change the nature of the solutions. We see that these solutions are excellent approximations to the numerical solutions (dashed lines in Fig. 3).

Whilst the solutions (7) do satisfy the given initial conditions, several were not formally imposed in the solution derivation. This is because the system (4) is singular as $\varepsilon \rightarrow 0$. Although we could formally match the solution (7) with an early-time solution, it is not useful for our eventual goal of determining 3HP production. Instead, we show that there are further early-time regions by Taylor expanding the initial conditions as $t\rightarrow 0^{+}$ in “Appendix A”. We see that these early-time solutions are excellent approximations to the numerical solutions (dash-dotted lines in Fig. 3).

#### Late time: $t= O (1/\varepsilon )$

The more interesting behaviour occurs for longer time. The solutions for $S_4$ and $R_2$, given in (7d) and (7h), respectively, appear to be unbounded. As we start with a finite level of nonreplenishable pyruvate, this is unphysical and suggests that there are further dynamics at play. We can obtain the correct scaling by looking for a change in leading-order terms in the system (4), which occurs when $t= O (1/\varepsilon )$. To investigate this, we introduce $t= T/ \varepsilon$, where $T= O (1)$, and we make the asymptotic scalings $(S_4, R_2) = \varepsilon ^{-1} (\overline{S}_4, \overline{R}_2)$, $(S_6, C_2, C_3, C_4) = \varepsilon (\overline{S}_6, \overline{C}_2, \overline{C}_3, \overline{C}_4)$, and $S_1= \varepsilon ^2 \overline{S}_1$. The long-term version of the system (4) is

9a $$\begin{aligned} \varepsilon \frac{\mathrm {d} \overline{S}_1}{\mathrm {d} T}&= - S_1- \varepsilon \bar{k}_{5}S_1S_4\left( 1 - \varepsilon ^4C_2\right) + \varepsilon \bar{k}_{-5}C_2+ \bar{k}_8C_4+ \varepsilon \bar{k}_{-8}\overline{S}_1R_1\left( 1 - \varepsilon ^3 C_4\right) , \end{aligned}$$

9b $$\begin{aligned} \frac{\mathrm {d} S_2}{\mathrm {d} T}&= \varepsilon S_1- \bar{k}_2S_2R_1, \end{aligned}$$

9c $$\begin{aligned} \varepsilon ^2 \frac{\mathrm {d} S_3}{\mathrm {d} T}&= \varepsilon \bar{k}_2S_2R_1- \bar{k}_{3}S_3\left( 1 - \varepsilon ^2 C_1\right) + \bar{k}_{-3}C_1, \end{aligned}$$

9d $$\begin{aligned} \frac{\mathrm {d} \overline{S}_4}{\mathrm {d} T}&= \bar{k}_4C_1- \varepsilon ^2 \bar{k}_{5}S_1S_4\left( 1 - \varepsilon ^4C_2\right) + \varepsilon ^2 \bar{k}_{-5}C_2, \end{aligned}$$

9e $$\begin{aligned} \frac{\mathrm {d} S_5}{\mathrm {d} T}&= \varepsilon ^2 \bar{k}_{6}C_2- \bar{k}_{7}S_5R_2\left( 1 - \varepsilon ^3 C_4\right) + \bar{k}_{-7}C_4, \end{aligned}$$

9f $$\begin{aligned} \varepsilon ^2 \frac{\mathrm {d} \overline{S}_6}{\mathrm {d} T}&= \bar{k}_{6}C_2- \bar{k}_{9}S_6\left( 1 - \varepsilon ^5 C_3\right) + \varepsilon \bar{k}_{-9}C_3, \end{aligned}$$

9g $$\begin{aligned} \frac{\mathrm {d} R_1}{\mathrm {d} T}&= - \bar{k}_2S_2R_1+ \varepsilon \bar{k}_8C_4- \varepsilon ^2 \bar{k}_{-8}\overline{S}_1R_1\left( 1 - \varepsilon ^3 \overline{C}_4\right) , \end{aligned}$$

9h $$\begin{aligned} \frac{\mathrm {d} \overline{R}_2}{\mathrm {d} T}&= \bar{k}_2S_2R_1- \varepsilon \bar{k}_{7}S_5R_2\left( 1 - \varepsilon ^3 C_4\right) + \varepsilon \bar{k}_{-7}C_4, \end{aligned}$$

9i $$\begin{aligned} \varepsilon ^3 \frac{\mathrm {d} C_1}{\mathrm {d} T}&=\bar{k}_{3}S_3\left( 1 - \varepsilon ^2 C_1\right) - \bar{k}_{-3}C_1- \varepsilon \bar{k}_4C_1, \end{aligned}$$

9j $$\begin{aligned} \varepsilon ^3 a_2\frac{\mathrm {d} \overline{C}_2}{\mathrm {d} T}&= \bar{k}_{5}S_1S_4\left( 1 - \varepsilon ^4C_2\right) - \bar{k}_{-5}C_2- \varepsilon \bar{k}_{6}C_2, \end{aligned}$$

9k $$\begin{aligned} \varepsilon ^3 a_3\frac{\mathrm {d} \overline{C}_3}{\mathrm {d} T}&= \bar{k}_{9}S_6\left( 1 - \varepsilon ^5 C_3\right) - \varepsilon \bar{k}_{-9}C_3- \bar{k}_{10}C_3, \end{aligned}$$

9l $$\begin{aligned} \varepsilon ^3 a_4\frac{\mathrm {d} \overline{C}_4}{\mathrm {d} T}&= \bar{k}_{7}S_5R_2\left( 1 - \varepsilon ^3 C_4\right) - \bar{k}_{-7}C_4- \bar{k}_8C_4+ \varepsilon \bar{k}_{-8}S_1R_1\left( 1 - \varepsilon ^3 C_4\right) , \end{aligned}$$

9m $$\begin{aligned} \frac{\mathrm {d} P}{\mathrm {d} T}&= \bar{k}_{10}C_3, \end{aligned}$$

and (5) becomes

9n $$\begin{aligned} \varepsilon \frac{\mathrm {d} S_3}{\mathrm {d} T} + \varepsilon ^2 \frac{\mathrm {d} C_1}{\mathrm {d} T}&= \bar{k}_2S_2R_1- \bar{k}_4C_1. \end{aligned}$$

The leading-order version of (9) is the differential-algebraic system

10a $$\begin{aligned}&\frac{\mathrm {d} S_2}{\mathrm {d} T} = - \bar{k}_2S_2R_1, \quad \frac{\mathrm {d} \overline{S}_4}{\mathrm {d} T} = \bar{k}_4C_1, \quad \frac{\mathrm {d} S_5}{\mathrm {d} T} = - \bar{k}_{7}S_5R_2+ \bar{k}_{-7}C_4, \quad \frac{\mathrm {d} R_1}{\mathrm {d} T} = - \bar{k}_2S_2R_1, \nonumber \end{aligned}$$

10b $$\begin{aligned}&\frac{\mathrm {d} \overline{R}_2}{\mathrm {d} T} = \bar{k}_2S_2R_1, \quad \frac{\mathrm {d} P}{\mathrm {d} T} = \bar{k}_{10}C_3, \\&S_1= \bar{k}_8C_4, \quad \bar{k}_{3}S_3= \bar{k}_{-3}C_1, \quad \bar{k}_{9}S_6= \bar{k}_{6}C_2, \quad \bar{k}_4C_1= \bar{k}_2S_2R_1, \quad \bar{k}_{-5}C_2= \bar{k}_{5}S_1S_4, \nonumber \\&\bar{k}_{10}C_3= \bar{k}_{9}S_6, \quad \left( \bar{k}_{-7}+ \bar{k}_8\right) C_4= \bar{k}_{7}S_5R_2. \end{aligned}$$

Now the system is controlled by the nonlinear dynamics of $S_2$ and $R_1$, and the solutions to the leading-order system (10) are

11a $$\begin{aligned} \overline{S}_1&= \beta _4\beta _5\varOmega \dfrac{ e^{\bar{k}_2(\alpha - 1)T} - 1}{\alpha e^{\bar{k}_2(\alpha - 1)T} - 1} \left( \dfrac{e^{-\alpha \bar{k}_2T} - \alpha e^{- \bar{k}_2T}}{1 - \alpha } \right) ^{\varOmega }, \end{aligned}$$

11b $$\begin{aligned} S_2&= \dfrac{\alpha - 1}{\alpha e^{\bar{k}_2(\alpha - 1)T} - 1}, \end{aligned}$$

11c $$\begin{aligned} S_3&= \beta _3(\alpha - 1)^2\dfrac{ e^{\bar{k}_2(\alpha - 1)T}}{(\alpha e^{\bar{k}_2(\alpha - 1)T} - 1)^2}, \end{aligned}$$

11d $$\begin{aligned} \overline{S}_4&= \alpha \dfrac{ e^{\bar{k}_2(\alpha - 1)T} - 1}{\alpha e^{\bar{k}_2(\alpha - 1)T} - 1}, \end{aligned}$$

11e $$\begin{aligned} S_5&= \beta _5\left( \dfrac{e^{-\alpha \bar{k}_2T} - \alpha e^{- \bar{k}_2T}}{1 - \alpha } \right) ^{\varOmega }, \end{aligned}$$

11f $$\begin{aligned} \overline{S}_6&= \alpha \beta _5\beta _6\varOmega \left( \dfrac{ e^{\bar{k}_2(\alpha - 1)T} - 1}{\alpha e^{\bar{k}_2(\alpha - 1)T} - 1}\right) ^2 \left( \dfrac{e^{-\alpha \bar{k}_2T} - \alpha e^{- \bar{k}_2T}}{1 - \alpha } \right) ^{\varOmega }, \end{aligned}$$

11g $$\begin{aligned} R_1&= \alpha (\alpha - 1)\dfrac{ e^{\bar{k}_2(\alpha - 1)T}}{\alpha e^{\bar{k}_2(\alpha - 1)T} - 1}, \end{aligned}$$

11h $$\begin{aligned} \overline{R}_2&=\alpha \dfrac{ e^{\bar{k}_2(\alpha - 1)T} - 1}{\alpha e^{\bar{k}_2(\alpha - 1)T} - 1}, \end{aligned}$$

11i $$\begin{aligned} C_1&= \gamma _1(\alpha - 1)^2\dfrac{ e^{\bar{k}_2(\alpha - 1)T}}{(\alpha e^{\bar{k}_2(\alpha - 1)T} - 1)^2}, \end{aligned}$$

11j $$\begin{aligned} \overline{C}_2&= \alpha \beta _5\beta _6\gamma _2\varOmega \left( \dfrac{ e^{\bar{k}_2(\alpha - 1)T} - 1}{\alpha e^{\bar{k}_2(\alpha - 1)T} - 1}\right) ^2 \left( \dfrac{e^{-\alpha \bar{k}_2T} - \alpha e^{- \bar{k}_2T}}{1 - \alpha } \right) ^{\varOmega }, \end{aligned}$$

11k $$\begin{aligned} \overline{C}_3&= \alpha \beta _5\gamma _3\varOmega \left( \dfrac{ e^{\bar{k}_2(\alpha - 1)T} - 1}{\alpha e^{\bar{k}_2(\alpha - 1)T} - 1}\right) ^2 \left( \dfrac{e^{-\alpha \bar{k}_2T} - \alpha e^{- \bar{k}_2T}}{1 - \alpha } \right) ^{\varOmega }, \end{aligned}$$

11l $$\begin{aligned} \overline{C}_4&= \beta _4\beta _5\varOmega \dfrac{ e^{\bar{k}_2(\alpha - 1)T} - 1}{\alpha e^{\bar{k}_2(\alpha - 1)T} - 1} \left( \dfrac{e^{-\alpha \bar{k}_2T} - \alpha e^{- \bar{k}_2T}}{1 - \alpha } \right) ^{\varOmega }, \end{aligned}$$

11m $$\begin{aligned} P&= \dfrac{\lambda \omega }{2 \left( \omega + 1\right) } + \dfrac{\alpha \beta _5\lambda \varOmega }{\bar{k}_2\left( 1 - \alpha \right) ^{\varOmega }} \int _0^{\bar{k}_2T} \! \left( e^{-\alpha s} - e^{-s} \right) ^2 \left( e^{-\alpha s} - \alpha e^{-s} \right) ^{\varOmega -2} \, \mathrm {d}s, \end{aligned}$$

where $\varOmega = \bar{k}_{7}\bar{k}_8/(\bar{k}_2(\bar{k}_{-7}+ \bar{k}_8))$, which is proportional to the initial concentration of ALT, the enzyme controlling (1e). Thus, $\varOmega$ is associated with the reversible reaction between l-alanine and pyruvate, and terms that are raised to the power of $\varOmega$ arise due to this reaction. In contrast, the terms not raised to the power of $\varOmega$ in the integrand of (11m) arise due to the conversion of pyruvate into $\beta$-alanine.

To match the long-term solutions (11) with the $t = O (1)$ solutions (7), we would have to consider intermediate matching regions where exponentially decreasing terms balance algebraically growing terms. These regions occur between $t = O (1)$ and $t = O (1/\varepsilon )$, and thus generally involve a translation in time of some multiple of $\log (1 / \varepsilon )$. As these regions are uninteresting—the decreasing and increasing terms pass by each other without interacting—we omit further discussion of these regions.

The long-time dynamics described by (11) are sensitive to the sign of $\alpha - 1$, with the singularities as $\alpha \rightarrow 1$ being removable. Moreover, the steady states in (11) as $T\rightarrow \infty$ depend on the sign of $\alpha - 1$, and thus are sensitive to the initial ratio of pyruvate to GLU. The exponential decay to these steady states slows down as $\alpha \rightarrow 1$, and becomes algebraic decay in the limit.

There is further long-time behaviour that can occur in this system, though only for certain parameter regimes. We discuss this behaviour in “Appendix B.1”.

### Discussion

Of the reactions given in (1), (1c–f) are heterologous in *Saccharomyces cerevisiae* (that is, they are not native to the microorganism), and are the most appropriate targets for enhancement. Thus, identifying and enabling the correct enzymes within cells for these reactions is paramount. To this end, we discuss our results in the context of being able to vary parameters from reactions (1c–f).

The general goal of the metabolic system we have considered is to maximise the total 3HP produced, whilst minimizing the malonic semialdehyde ($S_6$) produced, as the latter is toxic. The total 3HP produced at leading order, $P_{\text {tot}}$, can be obtained by taking the limit as $t\rightarrow \infty$ in (7m), and is given by

12a $$\begin{aligned} P_{\text {tot}}&= \lambda \left( \dfrac{ \omega }{2 \left( \omega + 1\right) } + \dfrac{\beta _5}{\bar{k}_2} I(\alpha ,\varOmega ) \right) , \end{aligned}$$

12b $$\begin{aligned} I(\alpha ,\varOmega )&= \dfrac{\alpha \varOmega }{\left( 1 - \alpha \right) ^{\varOmega }} \int _0^{\infty } \! \left( e^{-\alpha s} - e^{-s} \right) ^2 \left( e^{-\alpha s} - \alpha e^{-s} \right) ^{\varOmega -2} \, \mathrm {d}s. \end{aligned}$$

**Fig. 4:**
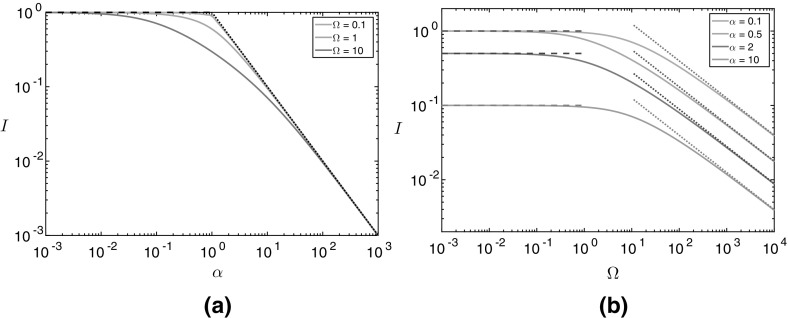
The function $I(\alpha ,\varOmega )$ defined in (12b). **a** Constant $\varOmega$ and varying $\alpha$. **b** Constant $\alpha$ and varying $\varOmega$. The solid curves are the numerically derived values, and the dashed and dotted curves are the asymptotic approximations for the small and large varying parameter, respectively. These approximations are defined in the text immediately below (12)

The integral (12b) has asymptotic behaviour $I \sim 1$ as $\alpha \rightarrow 0$, $I \sim 1/\alpha$ as $\alpha \rightarrow \infty$, $I \sim 1$ as $\varOmega \rightarrow 0$ for $\alpha < 1$, $I \sim 1/\alpha$ as $\varOmega \rightarrow 0$ for $\alpha > 1$, and $I \sim \sqrt{\pi /(2 \alpha \varOmega )}$ as $\varOmega \rightarrow \infty$, which agree well with the numerical solutions of (12b) (Fig. 4). To numerically evaluate (12b), we make the substitution $u = e^{- s}$ to switch to a finite domain and use an asymptotic approximation to evaluate the integral near $u=0$, where the integrand has an integrable singularity.

The amount of $S_6$ present in the system is at its maximum when $t= O (1)$, and we give an analytic expression for this in (7f), from which we see that

13a $$\begin{aligned} \max _{t>0} S_6(t)&= \beta _6f(\omega ;u(\omega )), \end{aligned}$$

13b $$\begin{aligned} f(\omega ;u(\omega ))&= \left( u + \dfrac{\omega ^2 e^{-u} - e^{-\omega u} + 1 - \omega ^2}{\omega (\omega -1)} \right) e^{-u}, \end{aligned}$$

where $$u(\omega ) > 0$$ satisfies

13c $$\begin{aligned} \dfrac{1 + 2 \omega }{\omega } + \dfrac{1 + \omega }{\omega (\omega - 1)}e^{-\omega u} = u + \dfrac{2 \omega }{\omega - 1} e^{-u}. \end{aligned}$$

**Fig. 5:**
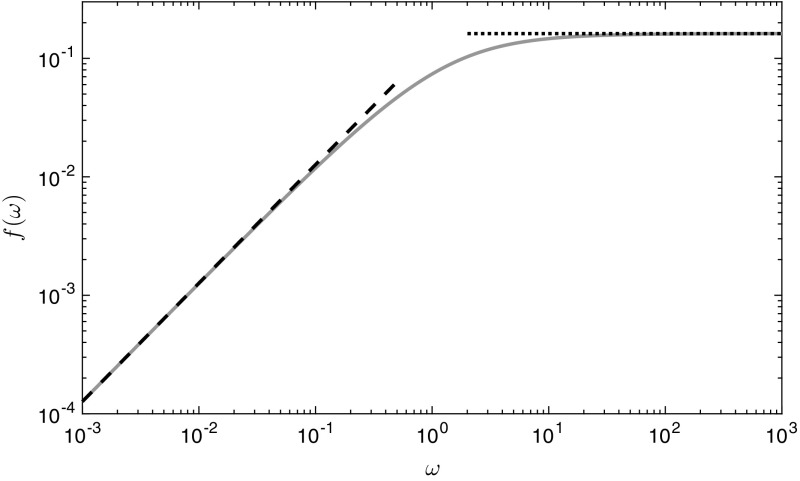
The function $f(\omega ;u(\omega ))$ defined in (13) is given by the grey curve. The dashed and dotted black curves are the small and large $\omega$ approximations, respectively. These approximations are defined in the text immediately below (13)

The function *f* is monotonically increasing in $\omega$, and bounded above (Fig. 5). For small $\omega$, we find that $f \sim \omega u^2 e^{-u}/4 \approx 0.127 \omega$ where *u* satisfies $e^{-u} = 1 - u + u^2 /4$ (thus, $u \approx 2.56$). For large $\omega$, we find that $f \sim u e^{-u}/2 \approx 0.162$ where *u* satisfies $e^{-u} = 1 - u/2$ (thus, $u \approx 1.59$). The numerical approximations we give are all to three significant figures.

From the solutions for the total 3HP produced, (12a), and the maximum value of malonic semialdehyde, (13), we see that increasing $\omega$ will increase both metabolites, but there is a limiting effect. Increasing $\omega$ corresponds to increasing the reaction rate through (1c), from aspartate to $\beta$-alanine, and thus we can deduce that this reaction step is important to our goals, but there are diminishing returns.

There is a greater effect on the system from the pre-factor constants $\lambda$ and $\beta _6= \lambda /\bar{k}_{9}$ for 3HP and malonic semialdehyde, respectively. $\lambda$ is a measure of the reaction rate through (1d), from $\beta$-alanine to malonic semialdehyde and l-alanine, and we can deduce that increasing the flux through this reaction will result in higher levels of 3HP and malonic semialdehyde. Moreover, a larger value of $\bar{k}_{9}$ will result in a lower maximum value of malonic semialdehyde, without affecting the levels of 3HP at leading order. This physically corresponds to choosing or designing an enzyme to which malonic semialdehyde will bind very quickly in the reaction (1f) from malonic semialdehyde to 3HP. Moreover, we see that increasing $\lambda$, and hence increasing the reaction rate through (1d), increases the levels of both 3HP and malonic semialdehyde without diminishing returns. Thus, (1d) is an important reaction in the system with regards to our goal, and the extra malonic semialdehyde produced by higher reaction rates through this system could be balanced by increasing $\bar{k}_{9}$.

The reaction (1e), where l-alanine reacts with AKG to produce pyruvate and GLU, and *vice versa*, is important for the long-time production of 3HP. The reversibility of this reaction means that the initial pyruvate is stored as l-alanine, then is able to be converted back into pyruvate due to the excess AKG produced by the reaction (1b), from oxaloacetate and GLU to aspartate and AKG. This process provides more pyruvate for the important reaction (1d), allowing it to proceed for a longer time. Bypassing the long route to $\beta$-alanine from pyruvate is important for this system, as the pyruvate runs out whilst the levels of $\beta$-alanine tend to a finite value. Thus, diverting pyruvate from producing to reacting with $\beta$-alanine will result in a more efficient system and thus increase the total 3HP produced. This effect manifests in the second term in (12a), where we find that increasing $\beta _5$, a measure of the backwards reaction in (1e), from pyruvate to l-alanine, increases the total 3HP produced for the reasons stated above. However, as the enzyme ALT used in this reaction also affects the value of $\varOmega$, care must be taken in interpreting this result. In particular, a large value of $\varOmega$ will reduce the amount of 3HP produced (Fig. 4).

**Fig. 6:**
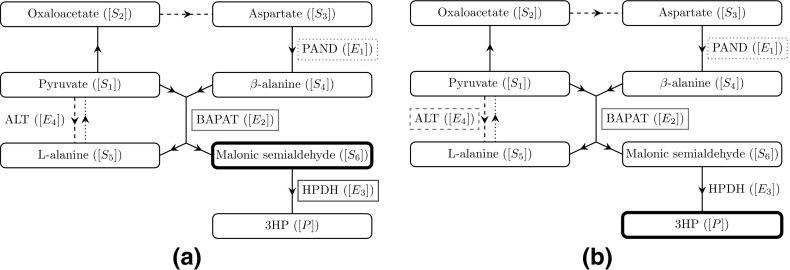
A schematic to highlight the effect of over-expressing a given enzyme in the no replenishment case on **a** malonic semialdehyde and **b** 3HP. The underlying network and the arrows between the nodes are explained in Fig. 1. An enzyme that is boxed and red/green means that over-expressing this enzyme causes a/an decrease/increase in the metabolite of interest. Our goal is to reduce the levels of malonic semialdehyde whilst increasing the levels of 3HP, where possible. A dashed box denotes that the over-expression has diminishing returns with *no* upper bound, and a dotted box denotes that the over-expression has diminishing returns *with* an upper bound (colour figure online)

With regards to over- or under-expressing enzymes in the system, we can use Table 2 to determine that $\omega \sim a_1$, $\lambda \sim a_2$, $\beta _5\sim a_4$, $\beta _6\sim a_2/ a_3$, and $\varOmega \sim a_4$, where $a_1$, $a_2$, $a_3$, and $a_4$ relate to the initial concentrations of the enzymes PAND, BAPAT, HPDH, and ALT, respectively, in the system. Hence, using the arguments presented above, we may also deduce the following results for enzyme expression (a schematic of these results is given in Fig. 6). Over-expressing PAND will monotonically increase the levels of both 3HP and the maximum malonic semialdehyde present, but the effect of this over-expression has diminishing returns and is bounded above. Similarly, over-expressing BAPAT will also result in higher levels of both 3HP and the maximum malonic semialdehyde present, but now without diminishing returns, as both scale linearly with $a_2$. Over-expressing HPDH decreases the maximum level of malonic semialdehyde present (scaling with $1/a_3$), but has no leading-order effect on the levels on 3HP. Finally, over-expressing ALT will increase the amount of 3HP produced (scaling with $\sqrt{a_4}$ for large $a_4$), without affecting the maximum (leading-order) level of malonic semialdehyde present. Thus, the effect of over-expressing ALT does have diminishing returns, but is not bounded above.

## Continuously replenished pyruvate

### Numerical results

**Fig. 7:**
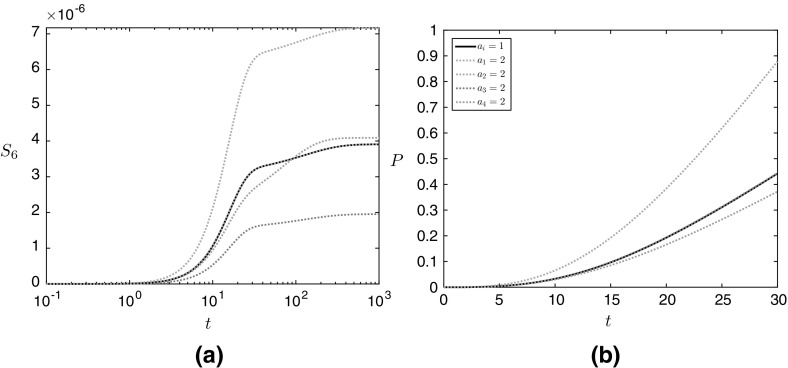
Numerical solutions in the continuously replenished pyruvate case for the dynamic concentrations of **a** malonic semialdehyde and **b** 3HP. We use the parameter values given in Table 2 for all simulations, and initial dimensionless enzyme concentrations of 1 unless specified in the legend. The solid black line corresponds to the reference simulation where $a_i = 1$ for $i = 1, 2, 3, 4$

We now investigate the system (4b–m), setting $S_1\equiv 1$. Thus, the concentration of pyruvate is no longer governed by (4a). This models a system where pyruvate is being constantly replenished and maintained at a given value. Solving this system numerically, we see that there are two main timescales for 3HP production, where $t= O (1)$ and $t= O (10^1)$, respectively (Fig. 7). In the $t= O (1)$ timescale, the levels of both malonic semialdehyde and 3HP are increasing, before the $t= O (10^1)$ timescale, where the levels of malonic semialdehyde increases at a much slower rate, and the levels of 3HP tends to a constant production rate. There is a further timescale when $t= O (10^2)$, and the levels of malonic semialdehyde slightly increase to a steady state in this timescale.

As with the never-replenished pyruvate case, the levels of malonic semialdehyde and 3HP can vary significantly when different enzymes are over-expressed. We show a schematic for the metabolic network in Fig. 1. Increasing the initial amount of PAND has no discernible effect on the levels of both malonic semialdehyde and 3HP. In contrast, over-expressing BAPAT significantly increases the levels of both malonic semialdehyde and 3HP. We see that over-expressing HPDH does not appear to have any effect on the levels of 3HP and, in fact, significantly decreases the maximum amount of malonic semialdehyde in the system. Finally, we see that over-expressing ALT has a small positive effect on the levels of malonic semialdehyde overall (though a small negative effect over $t= O (10^{1})$), and a small negative effect on the levels of 3HP.

We proceed in the same manner as §3, using an asymptotic analysis to explore the system (4b–m), setting $S_1\equiv 1$. This will allow us to determine how the system depends on the dimensionless parameters, and thus to explore the experimental parameter space without resorting to computationally expensive parameter sweeps.

### Asymptotic structure

As we did with §3, we now consider this system by exploiting the small parameter, $\varepsilon$, and using an asymptotic analysis. There are two main asymptotic regions of interest in time, where $t= O (1)$ and $t= O (1/\varepsilon ^{1/2})$, and we refer to these as medium and intermediate time, respectively (Fig. 7).

When $t= O (1)$, the system is governed by the release of pyruvate through the main pathway to 3HP and towards the production of l-alanine. There is a significant increase in the levels of all metabolites apart from pyruvate and GLU, the only metabolites that are initially present. When $t= O (1/\varepsilon ^{1/2})$, the initial rate of metabolite production is slowed and most metabolites approach their steady state. There is a further asymptotic region when $t= O (1/\varepsilon )$, and the system responds to the overproduction of l-alanine due to the continuous replenishment of pyruvate. We consider this region in “Appendix B.2 ”, as 3HP production is not affected by this redistribution. In the next section, we solve the leading-order versions of the system (4b–m), with $S_1\equiv 1$ in these asymptotic regions.

### Asymptotic solutions

The early-time behaviour of the system is the same as for the never-replenished case, and this is given in “Appendix A”. The more interesting behaviour for malonic semialdehyde and 3HP production occurs over longer timescales, which we now discuss in turn.

#### Medium time: $t= O (1)$

The leading-order system (4b–m), with $S_1\equiv 1$, as $\varepsilon \rightarrow 0$ is given by the differential-algebraic system

14a $$\begin{aligned}&\dot{S_2} = 1, \quad \dot{S_3} = \bar{k}_2S_2R_1- \bar{k}_4C_1, \quad \dot{S_4} = \bar{k}_4C_1, \quad \dot{S_5} = \bar{k}_{-7}C_4, \quad \dot{R_1} = 0, \nonumber \\&\dot{R_2} = \bar{k}_2S_2R_1+ \bar{k}_{-7}C_4, \quad \dot{P} = \bar{k}_{10}C_3, \end{aligned}$$

14b $$\begin{aligned}&\bar{k}_{9}S_6= \bar{k}_{6}C_2, \quad \bar{k}_{-3}C_1=\bar{k}_{3}S_3, \quad \bar{k}_{-5}C_2= \bar{k}_{5}S_1S_4, \quad \bar{k}_{10}C_3= \bar{k}_{9}S_6, \nonumber \\&\left( \bar{k}_{-7}+ \bar{k}_8\right) C_4= \bar{k}_{-8}S_1R_1, \end{aligned}$$

on using (5). The solution to (14) is

15a $$\begin{aligned} S_2&= t, \end{aligned}$$

15b $$\begin{aligned} S_3&= \dfrac{\beta _3}{\omega } \left( e^{-\omega t} - 1 + \omega t\right) , \end{aligned}$$

15c $$\begin{aligned} S_4&= \dfrac{\beta _4}{\omega ^2} \left( 1 - \omega t+ \dfrac{\omega ^2 t^2}{2}- e^{-\omega t} \right) , \end{aligned}$$

15d $$\begin{aligned} S_5&= \beta _5t, \end{aligned}$$

15e $$\begin{aligned} S_6&= \dfrac{\beta _6}{\omega ^2} \left( 1 - \omega t+ \dfrac{\omega ^2 t^2}{2}- e^{-\omega t} \right) , \end{aligned}$$

15f $$\begin{aligned} R_1&= \alpha , \end{aligned}$$

15g $$\begin{aligned} R_2&= \beta _4\dfrac{t^2}{2} + \beta _5t, \end{aligned}$$

15h $$\begin{aligned} C_1&= \dfrac{\gamma _1}{\omega } \left( e^{-\omega t} - 1 + \omega t \right) , \end{aligned}$$

15i $$\begin{aligned} C_2&= \dfrac{\gamma _2}{\omega ^2} \left( 1 - \omega t+ \dfrac{\omega ^2 t^2}{2}- e^{-\omega t} \right) , \end{aligned}$$

15j $$\begin{aligned} C_3&= \dfrac{\gamma _3}{\omega ^2}\left( 1 - \omega t+ \dfrac{\omega ^2 t^2}{2}- e^{-\omega t} \right) , \end{aligned}$$

15k $$\begin{aligned} C_4&= \gamma _4, \end{aligned}$$

15l $$\begin{aligned} P&= \dfrac{\lambda }{\omega ^3}\left( e^{-\omega t} - 1 + \omega t- \dfrac{\omega ^2 t^2}{2} + \dfrac{\omega ^3 t^3}{6}\right) , \end{aligned}$$

where the parameters are defined in (8). The solutions (15) show excellent agreement with the numerical results (dashed lines in Fig. 8). As with the case considered in §3, we did not have to impose all of the initial conditions to determine (15). That is, there is an early-time solution as $t\rightarrow 0^{+}$. For early time, the diminishing pyruvate case considered in the previous section is equivalent to the constant pyruvate case we consider here. Thus, the early-time behaviour for this case is also given in “Appendix A” and shows excellent agreement with the numerical results (dashed-dotted lines for early time in Fig. 8).

**Fig. 8:**
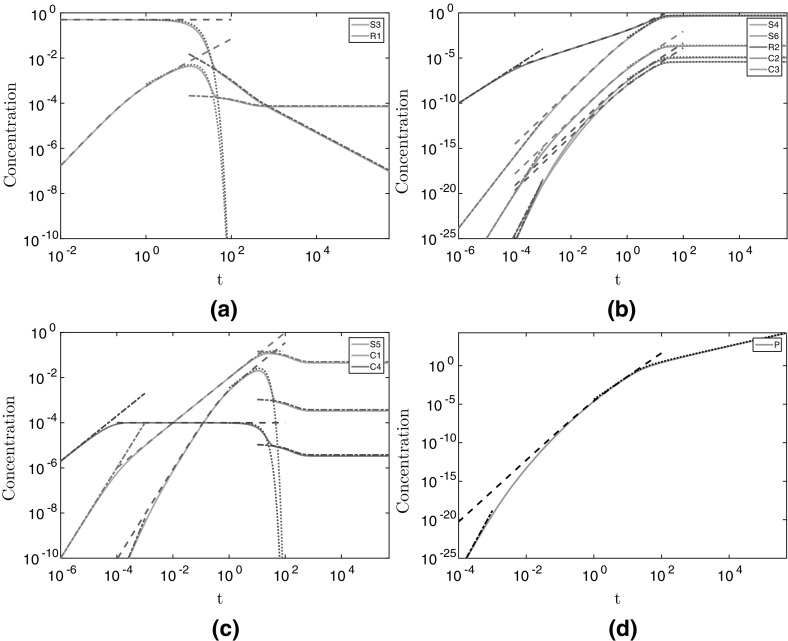
The numerical and asymptotic solutions for the metabolite concentrations in the continuously-replenished pyruvate case. The solid light lines denote the numerical solutions, and the broken darker lines denote the asymptotic solutions. Dash-dotted lines for $t < 10^{-3}$ represent the early-time solutions given in “Appendix A” (not shown in **a**), dashed lines represent the $t = O (1)$ solutions given in (15), dotted lines represent the intermediate-time solutions given in (18), and dash-dotted lines for $t > 10^{1}$ represent the late-time solutions given in (B7) (not shown for **c**, **d**). We use parameter values $\alpha = 0.5$, $a_1= 1, a_2= 1, a_3= 1$, and $a_4= 1$. We have split the metabolite concentrations into **a** metabolites whose early-time behaviour is described by the $t = O (1)$ behaviour, **b** metabolites whose late-time behaviour is described by the intermediate-time behaviour, **c** metabolites whose behaviour is distinct in each asymptotic region we have discussed, and **d** the product. We plot $([S_i],[R_i],[C_i],[P])/S_0$

#### Intermediate time: $t= O (1/\varepsilon ^{1/2})$

We can see that several terms in the system are promoted to leading order when $$t= O (\varepsilon ^{-1/2})$$. Using $$t= \tau /\varepsilon ^{1/2}$$ with the scalings $$(S_2, S_3, S_5, C_1) = (\hat{S}_2, \hat{S}_3, \hat{S}_5, \hat{C}_1)/\varepsilon ^{1/2}$$, $$(S_4, S_6, R_2, C_2, C_3) = (\hat{S}_4, \hat{S}_6, \hat{R}_2, \hat{C}_2, \hat{C}_3)/\varepsilon $$, and $$P= \hat{P}/ \varepsilon ^{3/2}$$, where all the new variables are $$ O (1)$$, the medium-time system (4) becomes

16a $$\begin{aligned} \frac{\mathrm {d} \hat{S}_2}{\mathrm {d} \tau }&= 1 - \varepsilon ^{1/2} \bar{k}_2\hat{S}_2R_1, \end{aligned}$$

16b $$\begin{aligned} \varepsilon ^{3/2} \frac{\mathrm {d} \hat{S}_3}{\mathrm {d} \tau }&= \varepsilon \bar{k}_2\hat{S}_2R_1-\bar{k}_{3}\hat{S}_3\left( 1 - \varepsilon ^{1/2} \hat{C}_1\right) + \bar{k}_{-3}\hat{C}_1, \end{aligned}$$

16c $$\begin{aligned} \frac{\mathrm {d} \hat{S}_4}{\mathrm {d} \tau }&= \bar{k}_4\hat{C}_1- \varepsilon ^{1/2} \bar{k}_{5}\hat{S}_4\left( 1 - \varepsilon ^2\hat{C}_2\right) + \varepsilon ^{1/2} \bar{k}_{-5}\hat{C}_2, \end{aligned}$$

16d $$\begin{aligned} \frac{\mathrm {d} \hat{S}_5}{\mathrm {d} \tau }&= \varepsilon \bar{k}_{6}\hat{C}_2- \varepsilon ^{1/2} \bar{k}_{7}\hat{S}_5\hat{R}_2\left( 1 - \varepsilon ^{2} C_4\right) + \bar{k}_{-7}C_4, \end{aligned}$$

16e $$\begin{aligned} \varepsilon ^{3/2} \frac{\mathrm {d} \hat{S}_6}{\mathrm {d} \tau }&= \bar{k}_{6}\hat{C}_2- \bar{k}_{9}\hat{S}_6\left( 1 - \varepsilon ^3 \hat{C}_3\right) + \varepsilon \bar{k}_{-9}\hat{C}_3, \end{aligned}$$

16f $$\begin{aligned} \frac{\mathrm {d} R_1}{\mathrm {d} \tau }&= - \bar{k}_2\hat{S}_2R_1+ \varepsilon ^{1/2} \bar{k}_8C_4- \varepsilon ^{1/2} \bar{k}_{-8}R_1\left( 1 - \varepsilon ^{2} C_4\right) , \end{aligned}$$

16g $$\begin{aligned} \frac{\mathrm {d} \hat{R}_2}{\mathrm {d} \tau }&= \bar{k}_2\hat{S}_2R_1- \varepsilon \bar{k}_{7}\hat{S}_5\hat{R}_2\left( 1 - \varepsilon ^{2} C_4\right) + \varepsilon ^{1/2} \bar{k}_{-7}C_4, \end{aligned}$$

16h $$\begin{aligned} \varepsilon ^{5/2} \frac{\mathrm {d} \hat{C}_1}{\mathrm {d} \tau }&=\bar{k}_{3}\hat{S}_3\left( 1 - \varepsilon ^{1/2} \hat{C}_1\right) - \bar{k}_{-3}\hat{C}_1- \varepsilon \bar{k}_4\hat{C}_1, \end{aligned}$$

16i $$\begin{aligned} \varepsilon ^{5/2} a_2\frac{\mathrm {d} \hat{C}_2}{\mathrm {d} \tau }&= \bar{k}_{5}\hat{S}_4\left( 1 - \varepsilon ^2\hat{C}_2\right) - \bar{k}_{-5}\hat{C}_2- \varepsilon \bar{k}_{6}\hat{C}_2, \end{aligned}$$

16j $$\begin{aligned} \varepsilon ^{5/2} a_3\frac{\mathrm {d} \hat{C}_3}{\mathrm {d} \tau }&= \bar{k}_{9}\hat{S}_6\left( 1 - \varepsilon ^3 \hat{C}_3\right) - \varepsilon \bar{k}_{-9}\hat{C}_3- \bar{k}_{10}\hat{C}_3, \end{aligned}$$

16k $$\begin{aligned} \varepsilon ^{5/2} a_4\frac{\mathrm {d} C_4}{\mathrm {d} \tau }&= \varepsilon ^{1/2} \bar{k}_{7}\hat{S}_5\hat{R}_2\left( 1 - \varepsilon ^{2} C_4\right) - \bar{k}_{-7}C_4- \bar{k}_8C_4+ \bar{k}_{-8}R_1\left( 1 - \varepsilon ^2 C_4\right) , \end{aligned}$$

16l $$\begin{aligned} \frac{\mathrm {d} \hat{P}}{\mathrm {d} \tau }&= \bar{k}_{10}\hat{C}_3, \end{aligned}$$

and (5) becomes

16m $$\begin{aligned} \varepsilon ^{1/2} \frac{\mathrm {d} \hat{S}_3}{\mathrm {d} \tau } + \varepsilon ^{3/2} \frac{\mathrm {d} \hat{C}_1}{\mathrm {d} \tau }&= \bar{k}_2\hat{S}_2R_1- \bar{k}_4\hat{C}_1. \end{aligned}$$

The leading-order version of (16) is given by the differential-algebraic system

17a $$\begin{aligned}&\frac{\mathrm {d} \hat{S}_2}{\mathrm {d} \tau } = 1, \quad \frac{\mathrm {d} \hat{S}_4}{\mathrm {d} \tau } = \bar{k}_4\hat{C}_1, \quad \frac{\mathrm {d} \hat{S}_5}{\mathrm {d} \tau } = \bar{k}_{-7}C_4, \quad \frac{\mathrm {d} R_1}{\mathrm {d} \tau } = - \bar{k}_2\hat{S}_2R_1, \quad \frac{\mathrm {d} \hat{R}_2}{\mathrm {d} \tau } = \bar{k}_2\hat{S}_2R_1, \nonumber \\&\frac{\mathrm {d} \hat{P}}{\mathrm {d} \tau } = \bar{k}_{10}\hat{C}_3, \end{aligned}$$

17b $$\begin{aligned}&\bar{k}_{3}\hat{S}_3= \bar{k}_{-3}\hat{C}_1, \quad \bar{k}_{9}\hat{S}_6= \bar{k}_{6}\hat{C}_2, \quad \bar{k}_4\hat{C}_1= \bar{k}_2\hat{S}_2R_1, \quad \bar{k}_{-5}\hat{C}_2= \bar{k}_{5}\hat{S}_4, \nonumber \\&\left( \bar{k}_{-7}+ \bar{k}_8\right) C_4= \bar{k}_{-8}R_1, \end{aligned}$$

and is solved by

18a $$\begin{aligned} \hat{S}_2&= \tau , \end{aligned}$$

18b $$\begin{aligned} \hat{S}_3&= \beta _3\tau e^{-\bar{k}_2\tau ^2 / 2}, \end{aligned}$$

18c $$\begin{aligned} \hat{S}_4&= \alpha \left( 1 - e^{-\bar{k}_2\tau ^2 / 2}\right) , \end{aligned}$$

18d $$\begin{aligned} \hat{S}_5&= \beta _5\sqrt{\dfrac{\pi }{2 \bar{k}_2}} {{\mathrm{erf}}}\left( \tau \sqrt{\dfrac{\bar{k}_2}{2}}\right) , \end{aligned}$$

18e $$\begin{aligned} \hat{S}_6&= \dfrac{\beta _6}{\bar{k}_2} \left( 1 - e^{-\bar{k}_2\tau ^2 / 2}\right) , \end{aligned}$$

18f $$\begin{aligned} R_1&= \alpha e^{-\bar{k}_2\tau ^2 / 2}, \end{aligned}$$

18g $$\begin{aligned} \hat{R}_2&= \alpha \left( 1 - e^{-\bar{k}_2\tau ^2 / 2}\right) , \end{aligned}$$

18h $$\begin{aligned} \hat{C}_1&= \gamma _1\tau e^{-\bar{k}_2\tau ^2 / 2}, \end{aligned}$$

18i $$\begin{aligned} \hat{C}_2&= \dfrac{\gamma _2}{\bar{k}_2} \left( 1 - e^{-\bar{k}_2\tau ^2 / 2}\right) , \end{aligned}$$

18j $$\begin{aligned} \hat{C}_3&= \dfrac{\gamma _3}{\bar{k}_2}\left( 1 - e^{-\bar{k}_2\tau ^2 / 2}\right) , \end{aligned}$$

18k $$\begin{aligned} C_4&= \gamma _4e^{-\bar{k}_2\tau ^2 / 2}, \end{aligned}$$

18l $$\begin{aligned} \hat{P}&= \dfrac{\lambda }{\bar{k}_2}\left( \tau - \sqrt{\dfrac{\pi }{2 \bar{k}_2}} {{\mathrm{erf}}}\left( \tau \sqrt{\dfrac{\bar{k}_2}{2}}\right) \right) , \end{aligned}$$

where the error function ${{\mathrm{erf}}}(z)$ is defined as

19 $$\begin{aligned} {{\mathrm{erf}}}(z) = \dfrac{2}{\sqrt{\pi }} \int _0^z \! e^{-s^2} \, \mathrm {d} s. \end{aligned}$$

These solutions show excellent agreement with the numerical results (dotted lines in Fig. 8).

Similarly to the analysis in §3, there is further long-time behaviour arising from higher-order terms becoming important as lower-order terms exponentially decrease. These can be obtained by proceeding to higher orders in the above analysis, but it is simpler to investigate the asymptotic balances in (16) using the solutions (18). As this does not affect the 3HP production at leading order, we discuss this in “Appendix B.2”.

### Discussion

We discuss our results in the same context as for the previous section. That is, we consider the effect of varying parameters from the heterologous reactions (1c–f), and with the goal of maximizing 3HP production whilst minimizing the maximum level of malonic semialdehyde, $S_6$.

We see from the long-time solution (18l) that 3HP ends up being produced at a constant rate $\lambda /\bar{k}_2$, and hence the concentration of 3HP ends up linearly increasing with time. Additionally, from (18e), the concentration of malonic semialdehyde monotonically increases and tends to a constant value of $\beta _6/\bar{k}_2= \lambda /(\bar{k}_2\bar{k}_{9})$. Hence, although there are clear differences in the long-time behaviour of this system compared to the never-replenished pyruvate case, there is a similar reaction dependence between systems. Specifically, whilst increasing the reaction rate through (1d), from $\beta$-alanine to malonic semialdehyde and l-alanine, will result in greater levels of 3HP and malonic semialdehyde, the levels of malonic semialdehyde produced can be reduced by increasing $\bar{k}_{9}$. That is, by selecting an enzyme to which malonic semialdehyde has a large binding affinity.

In contrast to the never-replenished pyruvate case, the continuously-replenished pyruvate case has no leading-order dependence on (1c), the reaction from aspartate to $\beta$-alanine, nor on (1e), the reaction where l-alanine reacts with AKG to produce pyruvate and GLU, and *vice versa*. Increasing the flux through the former and the reverse reaction of the latter had a positive effect on 3HP production, where the latter allowed l-alanine to initially act as a pyruvate store, before releasing pyruvate to react with $\beta$-alanine. Thus, for long-time, the rate of 3HP production and the levels of malonic semialdehyde are only dependent on the reaction (1d), compared to §3 where these quantities are dependent on (1c–e).

**Fig. 9:**
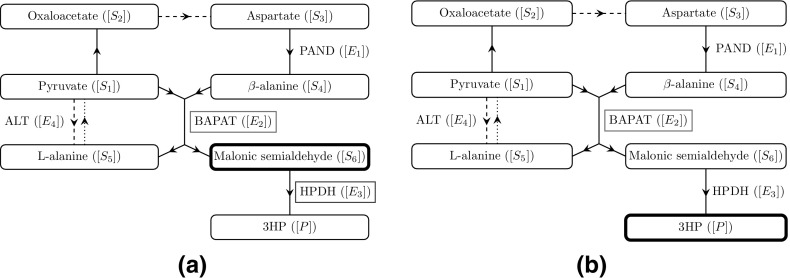
A schematic to highlight the effect of over-expressing a given enzyme in the continuous replenishment case on **a** malonic semialdehyde and **b** 3HP. The underlying network and the arrows between the nodes are explained in Fig. 1. An enzyme that is boxed and red/green means that over-expressing this enzyme causes a/an decrease/increase in the metabolite of interest. Our goal is to reduce the levels of malonic semialdehyde whilst increasing the levels of 3HP, where possible (colour figure online)

With regards to over- or under-expressing enzymes in the system, we can use Table 2 to determine that $\lambda \sim a_2$ and $\bar{k}_{9}\sim a_3$, where $a_2$ and $a_3$ relate to the initial concentrations of the enzymes BAPAT and HPDH, respectively, in the system (a schematic of these results is given in Fig. 9). Hence, using the arguments presented above, we may also deduce that over-expressing BAPAT will result in higher levels of both 3HP and the maximum malonic semialdehyde present, both scaling linearly with $a_2$. However, over-expressing HPDH decreases the maximum level of malonic semialdehyde present, which is inversely proportional to $a_3$, but has no leading-order effect on the levels on 3HP. Importantly, we also find that, in contrast to the non-replenished-pyruvate case, there is no leading-order dependence on the initial concentrations of PAND or ALT. Thus, our model suggests that the over-expression of PAND or ALT will not result in significant differences in the maximum level of malonic semialdehyde or the 3HP production for continuous replenishment of pyruvate.

## Conclusions

In this paper, we develop and solve a mathematical model for 3-hydroxypropionic acid (3HP) production from pyruvate via aspartate and $\beta$-alanine. We consider two limiting cases, where the initial level of pyruvate is never and continuously replenished, respectively. In both cases, we make substantial asymptotic progress to gain physical insight into the system behaviour, successfully comparing these results with numerical solutions to check their accuracy. Our asymptotic model (including relatively straightforward explicit expressions) allows us to predict and quantify ways to increase the levels of 3HP produced without increasing the levels of malonic semialdehyde, a toxic compound produced as an intermediate in the pathway.

**Fig. 10:**
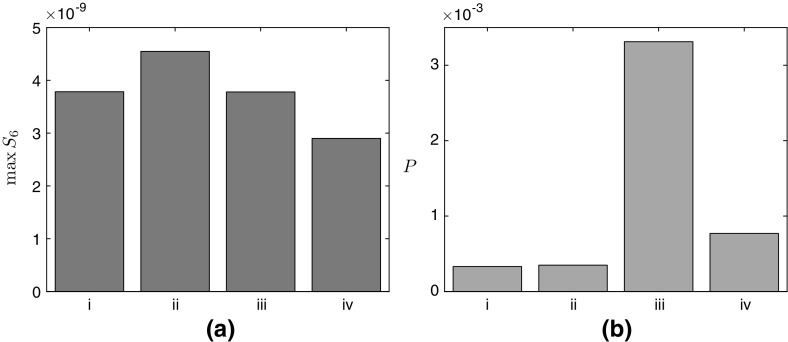
**a** The maximum level of malonic semialdehyde in the system, **b** the total 3HP produced, both in the no replenishment of pyruvate case. In both figures, the labels on the x-axis denote **i** Reference value (using the initial enzyme concentrations $a_1 = a_2 = a_3 = a_4 = 1$). (ii) Over-expressing PAND ($a_1 = 10, a_2 = a_3 = a_4 = 1$), (iii) Concurrently over-expressing BAPAT and HPDH ($a_1 = a_4 = 1$, $a_2 = a_3 = 10$), (iv) Over-expressing ALT ($a_1 = a_2 = a_3 = 1$, $a_4 = 10$). The units of the y-axis are $\mathrm {mM}$

**Fig. 11:**
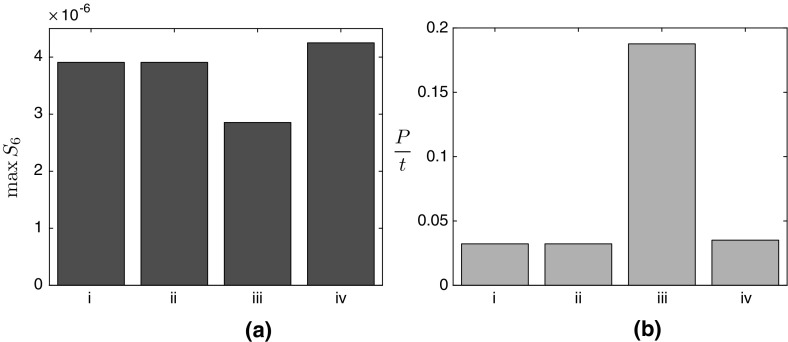
**a** The maximum level of malonic semialdehyde in the system, **b** the eventual rate of 3HP production, both in the continuous replenishment of pyruvate case. In **b**, we use *P* / *t* as the label for the *y*-axis as the levels of 3HP linearly increase with time (this continuous production is due to the continuous replenishment of pyruvate), and thus the appropriate measure here is the long-time production *rate* of 3HP. In both figures, the labels on the x-axis denote (i) Reference value (using the initial enzyme concentrations $$a_1 = a_2 = a_3 = a_4 = 1$$). (ii) Over-expressing PAND ($$a_1 = 10$$, $$ a_2 = a_3 = a_4 = 1$$), (iii) Concurrently over-expressing BAPAT and HPDH ($$a_1 = a_4 = 1$$, $$a_2 = a_3 = 10$$), (iv) Over-expressing ALT ($$a_1 = a_2 = a_3 = 1, a_4 = 10$$). The units of the y-axis are $$\mathrm {mM}$$

In terms of over-expressing enzymes, we find that for both cases the strongest positive effect comes from simultaneously over-expressing both BAPAT and HPDH. This significantly increase the levels of 3HP produced, but provides no significant increase in the maximum malonic semialdehyde present ((iii) in Figs. 10 and 11). Our results also show that over-expressing just one of BAPAT or HPDH will not have as strong an effect. We see a similar (though weaker) effect when over-expressing ALT for a limited supply of pyruvate, and the maximum level of malonic semialdehyde is slightly reduced ((iv) in Fig. 10). However, when pyruvate is continuously replenished, over-expressing ALT leads to a small increase in both the maximum level of malonic semialdehyde and the rate of 3HP production ((iv) in Fig. 11). Finally, over-expressing PAND only has a significant effect when there is a limited supply of pyruvate, where the over-expression leads to a slight increase in the maximum level of malonic semialdehyde present ((ii) in Fig. 10). This effect vanishes when the pyruvate is continuously replenished ((ii) in Fig. 11). We provide schematics outlining the effect of over-expressing a given enzyme on both malonic semialdehyde and 3HP in Figs. 6 and 9 for the never and continuous replenishment cases, respectively.

Another approach to optimizing metabolite pathways is to explore alternative enzymes for a given reaction. Our results can also be used to expedite this process, as we are able to determine how the system behaves as a function of the kinetic parameters of the enzymes, which will differ for alternative enzymes. Thus, we may interpret our results to determine the optimal targets for enzyme replacement. We find that, in both cases, the kinetic parameters involving the enzyme BAPAT ($\bar{k}_{5}$, $\bar{k}_{-5}$, and $\bar{k}_{6}$, defined in Table 2) have the most significant effect on 3HP production, and our model suggests that focusing attention on maximizing the parameter grouping $\bar{k}_{5}\bar{k}_{6}/ \bar{k}_{-5}$ will have the greatest effect on 3HP yield. Although increasing $\bar{k}_{5}\bar{k}_{6}/ \bar{k}_{-5}$ also has the effect of increasing the levels of malonic semialdehyde, we further show that choosing an HPDH enzyme to which malonic semialdehyde will quickly bind can negate this issue (that is, increasing $\bar{k}_{9}$, defined in Table 2), and we show in Figs. 10 and 11 that this combined effect of increasing the 3HP production whilst maintaining the maximum level of malonic semialdehyde can also be achieved by over-expressing BAPAT and HPDH.

We additionally show that the kinetic parameters involving the enzyme ALT ($\bar{k}_{7}$, $\bar{k}_{-7}$, $\bar{k}_8$, and $\bar{k}_{-8}$, defined in Table 2) have a leading-order effect on the 3HP levels when the initial level of pyruvate is never replenished. This is because the reversibility of the reaction effectively allows pyruvate to be stored and converted back when required. This storage effect is negligible in the continuous replenishment case as the pyruvate is plentiful (by definition) and thus there is no benefit to having additional storage of pyruvate. The important parameter groupings here are $\bar{k}_{7}\bar{k}_8/(\bar{k}_{-7}+ \bar{k}_8)$ and $\bar{k}_{-7}\bar{k}_{-8}/(\bar{k}_{7}\bar{k}_8)$, where increasing both of these dimensionless groupings leads to more 3HP produced, with no effect on the maximum levels of malonic semialdehyde present at leading order. We find that the combined effect of these parameters means that $\bar{k}_{-7}$ and $\bar{k}_{-8}$ should be increased, whereas $\bar{k}_{7}$ and $\bar{k}_8$ should be decreased, and we note that there are diminishing returns on increasing $\bar{k}_{-7}$. We show in Fig. 10 that this effect can be weakly obtained by over-expressing ALT.

The kinetic parameters of the remaining enzyme we consider, PAND, given by $\bar{k}_{3}$, $\bar{k}_{-3}$, and $\bar{k}_4$ defined in Table 2, are only significant at leading order in the never-replenished pyruvate case. In this case, we find that increasing the value of the parameter grouping $\bar{k}_{3}\bar{k}_4/ \bar{k}_{-3}$ increases both the amount of 3HP produced, and also the maximum levels of malonic semialdehyde present. However, the effect of increasing this parameter grouping is bounded above, and thus these diminishing returns suggest that this parameter grouping is unlikely to be particularly useful to exploit for the eventual goal of industrial production of 3HP.

We show that, although the full reaction dynamics are different between the never-replenished and continuously-replenished pyruvate cases, there are general similarities between predictions of the important reactions in both cases. Namely, our model suggests that over-expressing BAPAT or using a version of BAPAT with different kinetic parameters will increase levels of both 3HP and malonic semialdehyde, and that over-expressing HPDH or using an alternate that can very quickly bind to malonic semialdehyde will decrease the levels of malonic semialdehyde. The main differences between the two cases are as follows. Firstly, the kinetic parameters involved in the PAND and ALT reactions have a leading-order effect in the never-replenished pyruvate case, but not in the continuously-replenished case. Secondly, the time taken to reach the ‘long-time’ stage of 3HP production is asymptotically larger in the continuously-replenished pyruvate case, an effect relating to the continuous production of 3HP in this case. Thirdly, the malonic semialdehyde reaches its maximum value at a finite time in the never-replenished case, but tends to its maximum (constant) value in the continuously-replenished case.

We have made significant use of asymptotic analysis in this paper. This method has allowed us to determine the key parameter groupings involved in the production of 3HP and malonic semialdehyde. Moreover, there are some interesting aspects of the asymptotic analysis in its own right. For example, we have leading-order exponentially decreasing terms that become subdominant to algebraically increasing terms. Resolving this issue without going to higher asymptotic orders requires matching over a timescale that involves logarithmic functions of the small parameter $\varepsilon$, as shown in “Appendix B.1”. Another interesting facet of the asymptotic analysis we perform is that, in the never-replenished pyruvate case, there are effects that occur over two asymptotic timescales that contribute to the levels of 3HP at leading order, and the system behaviour over the longer timescale highlights how l-alanine acts as a store for pyruvate, yielding deeper physical insight into the system.

The two limiting cases of pyruvate replenishment that we have considered in this paper are chosen as modelling assumptions. In reality, the levels of pyruvate will fall somewhere between these two extremes, as some time-dependent level of pyruvate will be generated from glucose via glycolysis. Whilst our model does highlight the BAPAT enzyme as the most important to 3HP production in both of the extreme cases we consider, it may be interesting to investigate whether this is true for any given pyruvate production. This could be examined by imposing a certain time-dependent form for the pyruvate concentration (as we did by imposing that pyruvate was constant in the second case we considered) if it were known, or by including a known source of pyruvate in the governing equations and solving the full system. From (4), the governing equations for $$ O (1)$$ time, we can see that the leading-order metabolite concentrations can be written in terms of integrals of (time-dependent) imposed pyruvate concentrations or pyruvate sources, and these are given in “Appendix C”. It may be instructive to analysis these general results further.

Additionally, we chose somewhat arbitrary initial conditions which modelled the instantaneous addition of pyruvate to a well-mixed solution of enzymes. In reality, there is no clean starting point to such a set of reactions and there are likely to be small initial levels of every metabolite. We have tested our results for small but non-zero initial values of each metabolite and found that the early-time solutions we derived in “Appendix A” were inaccurate, but that the system relaxed into the remaining solutions we derived in this paper (results not shown).

Care must be taken with applying these results to *in vivo* experiments. Some of the metabolite concentrations we have derived are very small and, whilst this is not an issue for reactions occurring within a well-mixed beaker *in vitro*, it may pose a problem if the situation being modelled is reactions occurring within cells. For one molecule within a cell, the resulting macroscale concentration within a bacteria cell would be roughly $10^{-9} \, \mathrm {M} \approx \varepsilon ^3 S_0$ using the typical parameter values in this paper. Hence, care needs to be taken when interpreting our results for reactions occurring within bacterial cells. One option to accurately model a small number of molecules is is to perform stochastic simulations of the molecule numbers, assigning a probability of each reaction occurring. Additionally, for small numbers of molecules it may be that spatial effects are important. It would be useful to include these extra features in an extension of this work to check whether our conclusions still applied for *in vivo* experiments, but we note that this would significantly increase the computational expense of solving the model.

Finally, we note that this work highlights how mathematical models can be used to understand a complicated system, even one exhibiting nonlinear behaviour. From our model, we were able to highlight the strong positive effect of over-expressing two enzymes, BAPAT and HPDH, at the same time to increase 3HP production while maintaining the levels of malonic semialdehyde. Such theoretical results should allow significant reductions in the time taken to explore the experimental parameter space, and to aid in other ways the understanding of biological systems.

## Figures and Tables

**Fig. 12:**
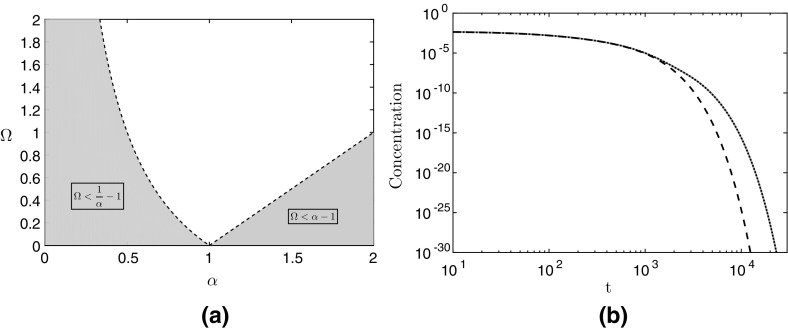
**a** The shaded areas denote the regions in which there is switching of asymptotic orders in the long-time solution for limited initial pyruvate. In the region where $$\varOmega < 1/\alpha - 1$$, solutions (B2) hold. In the region where $$\varOmega < \alpha - 1$$, solutions (B4) hold. **b** Results for $$S_3$$, taking $$\overline{k}_i = a_j = 1$$ for all *i*, *j*, and $$\alpha = 0.5$$, thus falling into the region where $$\alpha < 1$$ and $$\varOmega < 1/\alpha - 1$$. The solid grey curve shows the numerical result, the dashed black curve shows the $$ O (1)$$ term in (B2a), and the dotted black curve shows the full expression given in (B2a). The latter two asymptotic results show excellent agreement with the numerical result until $$t \approx 10^{3}$$, where asymptotic switching occurs, and the $$ O (\varepsilon )$$ term in (B2a) dominates

## Appendix A: Early-time solution

By Taylor expanding the system (4) around the initial conditions, we can deduce the leading-order behaviour

$$\begin{aligned} S_1&\sim 1, \quad S_2\sim t, \quad S_3\sim \alpha \bar{k}_2\dfrac{t^2}{2!}, \quad S_4\sim \dfrac{\alpha \bar{k}_2\bar{k}_{3}\bar{k}_4}{\varepsilon ^2}\dfrac{t^4}{4!}, \\ \quad S_5&\sim \dfrac{\alpha \bar{k}_{-7}\bar{k}_{-8}}{a_4\varepsilon ^2}\dfrac{t^2}{2!}, \quad S_6\sim \dfrac{\alpha \bar{k}_2\bar{k}_{3}\bar{k}_4\bar{k}_{5}\bar{k}_{6}}{a_2\varepsilon ^5}\dfrac{t^6}{6!}, \\ R_1&\sim \alpha , \quad R_2\sim \dfrac{\alpha \bar{k}_{-7}\bar{k}_{-8}}{a_4\varepsilon ^2}\dfrac{t^2}{2!}, \\ C_1&\sim \dfrac{\alpha \bar{k}_2\bar{k}_{3}}{\varepsilon ^2}\dfrac{t^3}{3!}, \quad C_2\sim \dfrac{\alpha \bar{k}_2\bar{k}_{3}\bar{k}_4\bar{k}_{5}}{ a_2\varepsilon ^4}\dfrac{t^5}{5!}, \quad C_3\sim \dfrac{\alpha \bar{k}_2\bar{k}_{3}\bar{k}_4\bar{k}_{5}\bar{k}_{6}\bar{k}_{9}}{ a_2a_3\varepsilon ^7}\dfrac{t^7}{7!}, \quad C_4\sim \dfrac{\alpha \bar{k}_{-8}}{a_4\varepsilon ^2}t, \\ P&\sim \dfrac{\alpha \bar{k}_2\bar{k}_{3}\bar{k}_4\bar{k}_{5}\bar{k}_{6}\bar{k}_{9}\bar{k}_{10}}{ a_2a_3\varepsilon ^7}\dfrac{t^8}{8!}, \nonumber \end{aligned}$$

as $t\rightarrow 0^{+}$. These results are expansions in which *t*, rather than $\varepsilon$, is the small parameter, and yield the earliest behaviour of the system. In Figs. 3 and 8, we see that these asymptotic solutions agree very well with the numerical solutions at early time. We note that these early-time solutions will be sensitive to any change in initial conditions.

## Appendix B: Long-time behaviour

In the main text, we have given the long-time behaviour of the system for times up to the asymptotic orders at which the levels of 3HP are affected. There is further interesting transient behaviour for the rest of the system and, in this Appendix, we discuss this behaviour.

### B.1 Never-replenished pyruvate

There is further long-time behaviour that can occur for the never-replenished pyruvate case which physically corresponds to a slower decay of metabolites than predicted in the main text. We see from the long-time solutions (11) that the metabolites that decay have decay rates of $|1 - \alpha |$ or $\min (\varOmega ,\alpha \varOmega )$. Thus, if higher order terms have slower decay rates than lower order terms in the long-time system (9), the asymptotic order of terms will eventually switch. We find that asymptotic switching occurs for $\alpha < 1$ if and only if $\varOmega < 1/\alpha - 1$, and for $\alpha > 1$ if and only if $\varOmega < \alpha - 1$ (Fig. 12a).

When $\alpha < 1$ and $\varOmega < 1/\alpha - 1$, we look into the region in (9) where

B1 $$\begin{aligned} T- \dfrac{\log (1/\varepsilon )}{\bar{k}_2(1 - \alpha - \alpha \varOmega )} = O (1), \end{aligned}$$

to determine the leading-order corrections

B2a $$\begin{aligned} S_3&\sim \beta _3(1 - \alpha )^2 e^{\bar{k}_2(\alpha - 1)T} + \varepsilon \dfrac{\beta _4\beta _5\varOmega \bar{k}_8}{\omega (1 - \alpha )^{\varOmega -1} (1 - \alpha - \alpha \varOmega )}e^{- \alpha \bar{k}_2\varOmega T}, \end{aligned}$$

B2b $$\begin{aligned} R_1&\sim \alpha (1 - \alpha ) e^{\bar{k}_2(\alpha - 1)T} + \varepsilon \dfrac{\beta _4\beta _5\varOmega \bar{k}_8}{\bar{k}_2(1 - \alpha )^\varOmega (1 - \alpha - \alpha \varOmega )}e^{- \alpha \bar{k}_2\varOmega T}, \end{aligned}$$

B2c $$\begin{aligned} C_1&\sim \gamma _1(1 - \alpha )^2 e^{\bar{k}_2(\alpha - 1)T} + \varepsilon \dfrac{\beta _4\beta _5\varOmega \bar{k}_8}{\bar{k}_4(1 - \alpha )^{\varOmega -1} (1 - \alpha - \alpha \varOmega )}e^{- \alpha \bar{k}_2\varOmega T}, \end{aligned}$$

as $$T\rightarrow \infty $$. The $$ O (\varepsilon )$$ terms in (B2) arise because the $$ O (1)$$ term has a larger exponential decay rate than the $$ O (\varepsilon )$$ term in (9g), and thus these terms will eventually balance. When $$\alpha >1$$ and $$\varOmega < \alpha - 1$$, we look into the region in (9) where

B3 $$\begin{aligned} T- \dfrac{\log (1/\varepsilon )}{\bar{k}_2(\alpha - 1 - \varOmega )} = O (1), \end{aligned}$$

to determine the leading-order corrections

B4a $$\begin{aligned} S_2&\sim \dfrac{\alpha - 1}{\alpha } e^{\bar{k}_2(1 - \alpha )T} + \varepsilon \left( \dfrac{\alpha }{\alpha - 1}\right) ^{\varOmega } \dfrac{\beta _5\varOmega }{ ( \alpha - 1 - \varOmega )} e^{- \bar{k}_2\varOmega T}, \end{aligned}$$

B4b $$\begin{aligned} S_3&\sim \beta _3\left( \dfrac{\alpha - 1}{\alpha }\right) ^2 e^{\bar{k}_2(1 - \alpha )T} + \varepsilon \left( \dfrac{\alpha }{\alpha - 1}\right) ^{\varOmega -1} \dfrac{\beta _4\beta _5\varOmega }{\omega ( \alpha - 1 - \varOmega )}e^{- \bar{k}_2\varOmega T}, \end{aligned}$$

B4c $$\begin{aligned} C_1&\sim \gamma _1\left( \dfrac{\alpha - 1}{\alpha }\right) ^2 e^{\bar{k}_2(1 - \alpha )T} + \varepsilon \left( \dfrac{\alpha }{\alpha - 1}\right) ^{\varOmega -1} \dfrac{\beta _4\beta _5\varOmega }{\bar{k}_4( \alpha - 1 - \varOmega )}e^{- \bar{k}_2\varOmega T}, \end{aligned}$$

as $$T\rightarrow \infty $$. The $$ O (\varepsilon )$$ terms in (B4) arise because the $$ O (1)$$ term has a larger exponential decay rate than the $$ O (\varepsilon )$$ term in (9b), and thus these terms will eventually balance. We see that these solutions are excellent approximations to the numerical solution.

This further long-time behaviour occurs when (1e), the reaction between l-alanine and pyruvate, is slow. The physical interpretation of the solutions in this appendix is that this slow reaction allows a slow replenishment of pyruvate which, in turn, is converted through the metabolites in the pathway to $\beta$-alanine. This slow release allows for a more gradual depletion of these intermediates.

### B.2 Continuously-replenished pyruvate

There is further long-time behaviour that can occur for the continuously-replenished pyruvate case which physically corresponds to some metabolites reaching a steady state rather than vanishing as predicted in the main text. Using $$\tau = T/\varepsilon ^{1/2}$$ (and hence $$t= T/\varepsilon $$) with the scalings $$(\hat{S}_2, \hat{P}) = \varepsilon ^{-1/2} (\tilde{S}_2, \tilde{P})$$, $$\hat{C}_4= \varepsilon ^{1/2} \tilde{C}_4$$, $$(\hat{S}_3, \hat{C}_1) = \varepsilon (\tilde{S}_3, \tilde{C}_1) $$, and $$R_1= \varepsilon ^{3/2} \tilde{R}_1$$, where all the new variables are $$ O (1)$$, the long-time system (16) becomes

B5a $$\begin{aligned} \frac{\mathrm {d} \tilde{S}_2}{\mathrm {d} T}&= 1 - \varepsilon ^{3/2} \bar{k}_2\tilde{S}_2\tilde{R}_1, \end{aligned}$$

B5b $$\begin{aligned} \varepsilon ^2 \frac{\mathrm {d} \tilde{S}_3}{\mathrm {d} T}&= \varepsilon \bar{k}_2\tilde{S}_2\tilde{R}_1-\bar{k}_{3}\tilde{S}_3\left( 1 - \varepsilon ^{3/2} \tilde{C}_1\right) + \bar{k}_{-3}\tilde{C}_1, \end{aligned}$$

B5c $$\begin{aligned} \frac{\mathrm {d} \hat{S}_4}{\mathrm {d} T}&= \varepsilon ^{1/2} \bar{k}_4\tilde{C}_1- \bar{k}_{5}\hat{S}_4\left( 1 - \varepsilon ^2\hat{C}_2\right) + \bar{k}_{-5}\hat{C}_2, \end{aligned}$$

B5d $$\begin{aligned} \frac{\mathrm {d} \hat{S}_5}{\mathrm {d} T}&= \varepsilon ^{1/2}\bar{k}_{6}\hat{C}_2- \bar{k}_{7}\tilde{S}_5\hat{R}_2\left( 1 - \varepsilon ^{5/2} \tilde{C}_4\right) + \bar{k}_{-7}\tilde{C}_4, \end{aligned}$$

B5e $$\begin{aligned} \varepsilon ^2 \frac{\mathrm {d} \hat{S}_6}{\mathrm {d} T}&= \bar{k}_{6}\hat{C}_2- \bar{k}_{9}\hat{S}_6\left( 1 - \varepsilon ^3 \hat{C}_3\right) + \varepsilon \bar{k}_{-9}\hat{C}_3, \end{aligned}$$

B5f $$\begin{aligned} \varepsilon \frac{\mathrm {d} \tilde{R}_1}{\mathrm {d} T}&= - \bar{k}_2\tilde{S}_2\tilde{R}_1+ \bar{k}_8\tilde{C}_4- \varepsilon \bar{k}_{-8}\tilde{R}_1\left( 1 - \varepsilon ^{5/2} \tilde{C}_4\right) , \end{aligned}$$

B5g $$\begin{aligned} \frac{\mathrm {d} \hat{R}_2}{\mathrm {d} T}&= \varepsilon ^{1/2} \bar{k}_2\tilde{S}_2\tilde{R}_1- \varepsilon ^{1/2} \bar{k}_{7}\tilde{S}_5\hat{R}_2\left( 1 - \varepsilon ^{5/2} \tilde{C}_4\right) + \varepsilon ^{1/2} \bar{k}_{-7}\tilde{C}_4, \end{aligned}$$

B5h $$\begin{aligned} \varepsilon ^{3} \frac{\mathrm {d} \tilde{C}_1}{\mathrm {d} T}&=\bar{k}_{3}\tilde{S}_3\left( 1 - \varepsilon ^{3/2} \tilde{C}_1\right) - \bar{k}_{-3}\tilde{C}_1- \varepsilon \bar{k}_4\tilde{C}_1, \end{aligned}$$

B5i $$\begin{aligned} \varepsilon ^{3} a_2\frac{\mathrm {d} \hat{C}_2}{\mathrm {d} T}&= \bar{k}_{5}\hat{S}_4\left( 1 - \varepsilon ^2\hat{C}_2\right) - \bar{k}_{-5}\hat{C}_2- \varepsilon \bar{k}_{6}\hat{C}_2, \end{aligned}$$

B5j $$\begin{aligned} \varepsilon ^{3} a_3\frac{\mathrm {d} \hat{C}_3}{\mathrm {d} T}&= \bar{k}_{9}\hat{S}_6\left( 1 - \varepsilon ^3 \hat{C}_3\right) - \varepsilon \bar{k}_{-9}\hat{C}_3- \bar{k}_{10}\hat{C}_3, \end{aligned}$$

B5k $$\begin{aligned} \varepsilon ^3 a_4\frac{\mathrm {d} \tilde{C}_4}{\mathrm {d} T}&= \bar{k}_{7}\hat{S}_5\hat{R}_2\left( 1 - \varepsilon ^{5/2} \tilde{C}_4\right) - \bar{k}_{-7}\tilde{C}_4- \bar{k}_8\tilde{C}_4+ \varepsilon \bar{k}_{-8}\tilde{R}_1\left( 1 - \varepsilon ^{5/2} \tilde{C}_4\right) , \end{aligned}$$

B5l $$\begin{aligned} \frac{\mathrm {d} \tilde{P}}{\mathrm {d} T}&= \bar{k}_{10}\hat{C}_3. \end{aligned}$$

To fully explain the long-term behaviour is it useful to consider some of the solutions up to $O (\varepsilon ^{1/2})$. Hence, we give (B5) up to $O (\varepsilon ^{1/2})$, resulting in the following differential-algebraic system

B6a $$\begin{aligned}&\frac{\mathrm {d} \tilde{S}_2}{\mathrm {d} T} =\! 1, \quad \frac{\mathrm {d} \hat{S}_4}{\mathrm {d} T} = \varepsilon ^{1/2} \bar{k}_4\tilde{C}_1- \bar{k}_{5}\hat{S}_4\!+\! \bar{k}_{-5}\hat{C}_2, \quad \frac{\mathrm {d} \hat{S}_5}{\mathrm {d} T} = \varepsilon ^{1/2} \bar{k}_{6}\hat{C}_2- \bar{k}_{7}\tilde{S}_5\hat{R}_2+ \bar{k}_{-7}\tilde{C}_4, \nonumber \\&\frac{\mathrm {d} \hat{R}_2}{\mathrm {d} T} = \varepsilon ^{1/2} \bar{k}_2\tilde{S}_2\tilde{R}_1- \varepsilon ^{1/2} \bar{k}_{7}\tilde{S}_5\hat{R}_2+ \varepsilon ^{1/2} \bar{k}_{-7}\tilde{C}_4, \quad \frac{\mathrm {d} \tilde{P}}{\mathrm {d} T} = \bar{k}_{10}\hat{C}_3, \end{aligned}$$

B6b $$\begin{aligned}&\bar{k}_{3}\tilde{S}_3= \bar{k}_{-3}\tilde{C}_1, \quad \bar{k}_{9}\hat{S}_6= \bar{k}_{6}\hat{C}_2, \quad \bar{k}_2\tilde{S}_2\tilde{R}_1= \bar{k}_8\tilde{C}_4, \quad \bar{k}_4\tilde{C}_1= \bar{k}_2\tilde{S}_2\tilde{R}_1, \quad \bar{k}_{-5}\hat{C}_2= \bar{k}_{5}\hat{S}_4, \nonumber \\&\bar{k}_{10}\hat{C}_3= \bar{k}_{9}\hat{S}_6, \quad \left( \bar{k}_{-7}+ \bar{k}_8\right) \tilde{C}_4= \bar{k}_{7}\hat{S}_5\hat{R}_2, \end{aligned}$$

on using (5) with the scalings introduced in this section.

The solution to (B6) is

B7a $$\begin{aligned} \tilde{S}_2&= T, \end{aligned}$$

B7b $$\begin{aligned} \tilde{S}_3&= \dfrac{1}{\omega } \left( \left( \beta _4\lambda \varOmega \sqrt{\dfrac{\pi }{2 \bar{k}_2}} - \varepsilon ^{1/2}\dfrac{\lambda }{\bar{k}_2} \right) e^{-\beta _4\varOmega T} + \varepsilon ^{1/2}\dfrac{\lambda }{\bar{k}_2} \right) , \end{aligned}$$

B7c $$\begin{aligned} \hat{S}_4&= \alpha , \end{aligned}$$

B7d $$\begin{aligned} \tilde{S}_5&= \dfrac{1}{\beta _4\varOmega } \left( \left( \beta _4\lambda \varOmega \sqrt{\dfrac{\pi }{2 \bar{k}_2}} - \varepsilon ^{1/2}\dfrac{\lambda }{\bar{k}_2} \right) e^{-\beta _4\varOmega T} + \varepsilon ^{1/2}\dfrac{\lambda }{\bar{k}_2} \right) , \end{aligned}$$

B7e $$\begin{aligned} \hat{S}_6&= \dfrac{\beta _6}{\bar{k}_2}, \end{aligned}$$

B7f $$\begin{aligned} \tilde{R}_1&= \dfrac{1}{\bar{k}_2T} \left( \left( \beta _4\lambda \varOmega \sqrt{\dfrac{\pi }{2 \bar{k}_2}} - \varepsilon ^{1/2}\dfrac{\lambda }{\bar{k}_2} \right) e^{-\beta _4\varOmega T} + \varepsilon ^{1/2}\dfrac{\lambda }{\bar{k}_2} \right) , \end{aligned}$$

B7g $$\begin{aligned} \hat{R}_2&= \alpha , \end{aligned}$$

B7h $$\begin{aligned} \tilde{C}_1&= \dfrac{1}{\bar{k}_4} \left( \left( \beta _4\lambda \varOmega \sqrt{\dfrac{\pi }{2 \bar{k}_2}} - \varepsilon ^{1/2}\dfrac{\lambda }{\bar{k}_2} \right) e^{-\beta _4\varOmega T} + \varepsilon ^{1/2}\dfrac{\lambda }{\bar{k}_2} \right) , \end{aligned}$$

B7i $$\begin{aligned} \hat{C}_2&= \dfrac{\gamma _2}{\bar{k}_2}, \end{aligned}$$

B7j $$\begin{aligned} \hat{C}_3&= \dfrac{\gamma _3}{\bar{k}_2}, \end{aligned}$$

B7k $$\begin{aligned} \tilde{C}_4&= \dfrac{1}{\bar{k}_8} \left( \left( \beta _4\lambda \varOmega \sqrt{\dfrac{\pi }{2 \bar{k}_2}} - \varepsilon ^{1/2}\dfrac{\lambda }{\bar{k}_2} \right) e^{-\beta _4\varOmega T} + \varepsilon ^{1/2}\dfrac{\lambda }{\bar{k}_2} \right) , \end{aligned}$$

B7l $$\begin{aligned} \tilde{P}&= \dfrac{\lambda }{\bar{k}_2} T, \end{aligned}$$

where we give the solutions up to $$ O (\varepsilon ^{1/2})$$ for the metabolites where the $$ O (1)$$ term is exponentially decreasing. In these cases, there is a constant $$ O (\varepsilon ^{1/2})$$ term, and we wish to avoid introducing a further boundary layer when $$T= (1/(2 \beta _4\varOmega )) \log (1/\varepsilon ) + O (1)$$ to switch the asymptotic order of terms. To give the $$ O (\varepsilon ^{1/2})$$ terms for the remaining metabolites, we would need to match with higher order terms from the $$t = O (\varepsilon ^{-1/2})$$ region which, in turn, we would need to match with higher order terms from the $$t = O (1)$$ region. As we are satisfied with our current level of accuracy, we omit this for brevity. The solutions (B7) show excellent agreement with the numerical results (dash-dotted lines for late time in Fig. 8).

The long-time behaviour (B7) allows the levels of aspartate and l-alanine to attain their correct steady state values which are limited by (1d), the BAPAT reaction. The levels of aspartate sharply decrease when $t= O (1/\varepsilon ^{1/2})$ as aspartate is converted into $\beta$-alanine which is free to react with all the excess pyruvate in the system. In (B7), this sharp decrease is balanced by a slow but steady conversion of pyruvate (through oxaloacetate) into aspartate, allowing for a finite steady state. The levels of l-alanine are artificially high when $t= O (1/\varepsilon ^{1/2})$ due to the reaction of $\beta$-alanine with all the excess pyruvate into l-alanine (and malonic semialdehyde). This large production of l-alanine is balanced in (B7) by the conversion of l-alanine into pyruvate.

## Appendix C: General solutions for $t= O (1)$

The general problem for $t= O (1)$ is given by (6), ignoring the equation $\dot{S_1} = -S_1$. Instead, $S_1$ is either imposed to be a specific function of time, or governed by

C1 $$\begin{aligned} \dot{S_1}&= f(t) - S_1, \end{aligned}$$

at leading order for small $\varepsilon$, where $f(t)$ represents a time-dependent source of pyruvate. In this paper, we have considered the cases $f \equiv 0$ and $S_1\equiv 1$ (equivalent to $f \equiv 1$ at leading order) in more detail, corresponding to no replenishment and continuous replenishment of pyruvate, respectively. As (C1) is solved by

C2 $$\begin{aligned} S_1= e^{-t} + \int _0^{t} \! e^{s - t} f(s) \, \mathrm {d} s, \end{aligned}$$

we can determine $S_1(t)$ in either case. Hence, we give the solution to (6) in terms of a general $S_1(t)$ (ignoring the equation $\dot{S_1} = - S_1$), as follows

C3a $$\begin{aligned} S_2&= \int _0^{t} \! S_1(s) \, \mathrm {d} s, \end{aligned}$$

C3b $$\begin{aligned} S_3&= \beta _3\int _0^{t} \! \left( 1 - e^{\omega (s - t)} \right) S_1(s) \, \mathrm {d}s, \end{aligned}$$

C3c $$\begin{aligned} S_4&= \beta _4\int _0^{t} \! \left( t- s + \dfrac{e^{\omega (s - t)} - 1}{\omega } \right) S_1(s) \, \mathrm {d}s, \end{aligned}$$

C3d $$\begin{aligned} S_5&= \beta _5\int _0^{t} \! S_1(s) \, \mathrm {d} s, \end{aligned}$$

C3e $$\begin{aligned} S_6&= \beta _6S_1(t) \int _0^{t} \! \left( t- s + \dfrac{e^{\omega (s - t)} - 1}{\omega } \right) S_1(s) \, \mathrm {d}s, \end{aligned}$$

C3f $$\begin{aligned} R_1&= \alpha , \end{aligned}$$

C3g $$\begin{aligned} R_2&= \int _0^{t} \! \left( \beta _4\left( t- s\right) + \beta _5\right) S_1(s) \, \mathrm {d}s, \end{aligned}$$

C3h $$\begin{aligned} C_1&= \gamma _1\int _0^{t} \! \left( 1 - e^{\omega (s - t)} \right) S_1(s) \, \mathrm {d}s, \end{aligned}$$

C3i $$\begin{aligned} C_2&= \gamma _2S_1(t) \int _0^{t} \! \left( t- s + \dfrac{e^{\omega (s - t)} - 1}{\omega } \right) S_1(s) \, \mathrm {d}s, \end{aligned}$$

C3j $$\begin{aligned} C_3&= \gamma _3S_1(t) \int _0^{t} \! \left( t- s + \dfrac{e^{\omega (s - t)} - 1}{\omega } \right) S_1(s) \, \mathrm {d}s, \end{aligned}$$

C3k $$\begin{aligned} C_4&= \gamma _4S_1(t), \end{aligned}$$

C3l $$\begin{aligned} P&= \lambda \int _0^{t} S_1(s) \int _0^{s} \! \left( s - v + \dfrac{e^{\omega (v - s)} - 1}{\omega } \right) S_1(v) \, \mathrm {d}v \, \mathrm {d}s. \end{aligned}$$

## References

[CR1] Albe KR, Butler MH, Wright BE (1990). Cellular concentrations of enzymes and their substrates. J Theor Biol.

[CR2] Berg IA, Kockelkorn D, Buckel W, Fuchs G (2007). A 3-hydroxypropionate/4-hydroxybutyrate autotrophic carbon dioxide assimilation pathway in archaea. Science.

[CR3] Borodina I, Kildegaard KR, Jensen NB, Blicher TH, Maury J, Sherstyk S, Schneider K, Lamosa P, Herrgård MJ, Rosenstand I, Öberga F, Forstera J, Nielsena J (2015). Establishing a synthetic pathway for high-level production of 3-hydroxypropionic acid in saccharomyces cerevisiae via $$\beta $$-alanine. Metab Eng.

[CR4] Branson JP, Nezic M, Wallace JC, Attwood PV (2002). Kinetic characterization of yeast pyruvate carboxylase isozyme pyc1. Biochemistry.

[CR5] Chopra S, Pai H, Ranganathan A (2002). Expression, purification, and biochemical characterization of mycobacterium tuberculosis aspartate decarboxylase, pand. Protein Express Purif.

[CR6] Hayaishi O, Nishizuka Y, Tatibana M, Takeshita M, Kuno S (1961). Enzymatic studies on the metabolism of $$\beta $$-alanine. J Biol Chem.

[CR7] Hinch EJ (1991). Perturbation methods.

[CR8] Jiang X, Meng X, Xian M (2009). Biosynthetic pathways for 3-hydroxypropionic acid production. Appl Microbiol Biotechnol.

[CR9] Jitrapakdee S, Adina-Zada A, Besant PG, Surinya KH, Cleland WW, Wallace JC, Attwood PV (2007). Differential regulation of the yeast isozymes of pyruvate carboxylase and the locus of action of acetyl coa. Int J Biochem Cell Biol.

[CR10] Kevorkian J, Cole JD (2013). Perturbation methods in applied mathematics.

[CR11] Kockelkorn D, Fuchs G (2009). Malonic semialdehyde reductase, succinic semialdehyde reductase, and succinyl-coenzyme a reductase from metallosphaera sedula: enzymes of the autotrophic 3-hydroxypropionate/4-hydroxybutyrate cycle in sulfolobales. J Bacteriol.

[CR12] Kumar V, Ashok S, Park S (2013). Recent advances in biological production of 3-hydroxypropionic acid. Biotechnol Adv.

[CR13] Nakano Y, Tokunaga H, Kitaoka S (1977). Two $$\omega $$-amino acid transaminases from *Bacillus cereus*. J Biochem.

[CR14] Nobe Y, Kawaguchi S, Ura H, Nakai T, Hirotsu K, Kato R, Kuramitsu S (1998). The novel substrate recognition mechanism utilized by aspartate aminotransferase of the extreme thermophile *Thermus thermophilus* hb8. J Biol Chem.

[CR15] O’Malley Jr RE (2012). Singular perturbation methods for ordinary differential equations.

[CR16] Ramjee MK, Genschel U, Abell C, Smith AG (1997). *Escherichia coli*l-aspartate-$$\alpha $$-decarboxylase: preprotein processing and observation of reaction intermediates by electrospray mass spectrometry. Biochem J.

[CR17] Umemura I, Yanagiya K, Komatsubara S, Sato T, Tosa T (1994). Purification and some properties of alanine aminotransferase from *Candida maltosa*. Biosci Biotechnol Biochem.

[CR18] Ward DE, Kengen SWM, van der Oost J, de Vos WM (2000). Purification and characterization of the alanine aminotransferase from the hyperthermophilic archaeon *Pyrococcus furiosus* and its role in alanine production. J Bacteriol.

[CR19] Werpy T, Petersen G, Aden A, Bozell J, Holladay J, White J, Manheim A, Eliot D, Lasure L, Jones S (2004) Top value added chemicals from biomass, volume 1-results of screening for potential candidates from sugars and synthesis gas. Technical report, DTIC Document

[CR20] Williamson JM, Brown GM (1979). Purification and properties of l-aspartate-alpha-decarboxylase, an enzyme that catalyzes the formation of beta-alanine in *Escherichia coli*. J Biol Chem.

[CR21] Yagi T, Kagamiyama H, Nozaki M (1982). Aspartate: 2-oxoglutarate aminotransferase from bakers’ yeast: crystallization and characterization. J Biochem.

